# Eradication of tumors and development of anti-cancer immunity using STINGa targeted by pHLIP

**DOI:** 10.3389/fonc.2022.1023959

**Published:** 2022-10-18

**Authors:** Anna Moshnikova, Michael DuPont, Hannah Visca, Donald M. Engelman, Oleg A. Andreev, Yana K. Reshetnyak

**Affiliations:** ^1^ Physics Department, University of Rhode Island, Kingston, RI, United States; ^2^ Department of Molecular Biophysics and Biochemistry, Yale, New Haven, CT, United States

**Keywords:** immunotherapy, tumor acidity, tumor pH, tumor stroma, targeted STINGa

## Abstract

Despite significant progress in the development of novel STING agonists (STINGa), applications appear to be challenged by the low efficiency and poor selectivity of these agents. A pH Low Insertion Peptide (pHLIP) extends the lifetime of a STINGa in the blood and targets it to acidic cancer-associated fibroblasts (CAFs), tumor-associated macrophages (TAMs), myeloid derived suppressor cells (mMDSCs) and dendritic cells (DCs). CAFs constitute 25% of all live cells within CT26 tumors, and M2-type TAMs and mMDSCs are the most abundant among the immune cells. The resulting activation of cytokines within the tumor microenvironment (TME) triggers the eradication of small (100 mm^3^) and large (400-700 mm^3^) CT26 tumors in mice after a single dose of pHLIP-STINGa. The tumor stroma was destroyed (the number of CAFs was reduced by 98%), intratumoral hemorrhage developed, and the level of acidity within the TME was reduced. Further, no tumors developed in 20 out of 25 tumor-free mice re-challenged by an additional injection of cancer cells. The therapeutic effect on CT26 tumors was insignificant in nude mice, lacking T-cells. Thus, targeted delivery of STINGa to tumor stroma and TAMs induces activation of signaling, potentially resulting in the recruitment and infiltration of T-cells, which gain access to the tumor core. The cytotoxic activity of T-cells is not impaired by an acidic environment and immune memory is developed.

## Introduction

Immune evasion is a hallmark of cancer. Overcoming that evasion to harness the power of the immune system to attack tumors has become a widely employed strategy (1, 2). In recent years, successful cancer treatments have been introduced that are based on the use of immune checkpoint inhibitors (ICIs) (3). The checkpoint blockade approach directly targets the adaptive immune system, acting to release the brakes on anti-tumor immune T-cells (4). However, only a limited variety of tumor types respond to this therapy. Unresponsive tumors are immunologically non-inflamed or “cold” tumors, exhibiting low cytokine expression and a lack of T-cell and NK-cell infiltration (5). Recently, immune stimulatory strategies have been introduced that are based on the activation of the innate immune system and the enhancement of tumor immunogenicity (6, 7). Activation of the stimulator of interferon genes (STING) pathway culminates in the initiation of interferon regulatory factor 3, NF-kappa-B-dependent transcriptional programs and autophagy (8). Using STING agonists (STINGa) to “heat up” the tumor microenvironment results in an anti-tumor immune response. Therefore, STINGa were developed that exhibited promising activity in preclinical animal models, and clinical trials were initiated (7). However, the poor pharmacokinetics of STINGa and systemic immuno-activation side effects required intra-tumoral dosing in most cases, significantly limiting applications. In general, the first clinical trials have resulted in disappointingly modest efficacy, but targeted delivery of STINGa might overcome the difficulties by allowing general administration without systemic immune-activation and improved pharmacokinetics (9). We have found that targeted delivery of STINGa to tumor stroma by pHLIP, which senses acidity at the surface of metabolically active cells (10), effectively stimulates an antitumor response with a single administration and immune memory is developed.

## Methods

### Synthesis of pHLIP-STINGa

A diABZI STING agonist was modified with a linker to prepare o-pyridyl-dithioethyl-carbamoyl-PAB-STING (Pys-PAB-STINGa). The agent was synthesized and purified by Iris Biotech GmbH. All pHLIP peptides used in the study were synthesized and purified at CSBio. For conjugation with Pys-PAB-STINGa the following pHLIP sequences with single Cys residues at the membrane-inserting ends of the peptides were used:

pHLIP(Laa): ADDQNPWRAYLDLLFPTDTLLLDLLWCG consisting of all L amino acids and
pHLIP(Daa): ADQDNPWRAYLDLLFPTDTLLLDLLWCG consisting of all D amino acids.

pHLIP peptides and Pys-PAB-STINGa were mixed in dimethyl sulfoxide (DMSO) at a molar ratio 1:1. Sodium phosphate buffer (100 mM) containing 150 mM NaCl at pH 7.4 saturated with argon was added to the reaction mix (1/10 of the total volume) and the reaction mixture was kept for 2 hours at room temperature (RT). pHLIP-STINGa constructs were purified by reverse phase high-performance liquid chromatography (HPLC) using Zorbax SB-C18, 9.4×250 mm, 5 μm column (Agilent Technology) with a gradient from 10% to 75% acetonitrile in water containing 0.05% of trifluoroacetic acid (TFA). For preparation of fluorescent versions of the agents, Al647-pHLIP-STINGa and ICG-pHLIP-STINGa, N-acetylated versions of the pHLIP peptide AKDDQNPWRAYLDLLFPTDTLLLDLLWCG consisting of all L amino acids was used. First, pHLIP was conjugated with Pys-PAB-STING, followed by purification. Then, either ICG-NHS ester (Iris Biotech GmbH) or Alexa647-NHS ester (Life Technologies) was conjugated with the lysine residue at the N-terminal end of pHLIP in DMSO at molar ratio of 1:1.5. Sodium bicarbonate buffer (100 mM) at pH 8.3 was added to the reaction mix (1/10 of the total volume) and the reaction mixture was kept at RT until the conjugation was completed. The final purification was performed as described above. The products were lyophilized and characterized by matrix-assisted laser desorption/ionization-time of flight (MALDI-TOF) mass-spectrometry and analytical HPLC. The concentration of pHLIP-STINGa conjugates was determined by absorbance using the following molar extinction coefficients: for pHLIP-STINGa *ε*_322_ = 46,200 M^−1^cm^−1^, for ICG-pHLIP-STINGa *ε*_800_ = 137,000 M^−1^cm^−1^ and for Al647-pHLIP-STINGa *ε*_651_ = 270,000 M^−1^cm^−1^.

### Stability in mouse and human plasma

To establish the stability of pHLIP(Laa)-STINGa and pHLIP(Daa)-STINGa in plasma, pHLIP-STINGa was mixed with single donor human or BALB/c mouse plasma (Innovative Research) at a concentration of 200 μM, and kept in plasma for 0, 2, 4 or 24 hours at 37°C. Plasma proteins were precipitated by methanol (1:5 volume ratio of plasma to methanol) and centrifugated for 10 min at 13.4 rpm. The supernatant was collected and analyzed by HPLC using a Zorbax SB-C18 4.6 × 250 mm, 5 μm column with a gradient from 10% to 75% acetonitrile in water containing 0.05% TFA. Chromatograms were recorded at 220 nm, 280 nm and 320 nm. As controls pHLIP(Laa), pHLIP(Daa), Pys-PAB-STINGa, SH-PAB-STINGa, where Pys-PAB-STINGa was conjugated with a Cys residue, and diABZI (*In vivo*gen) were analyzed for stability in plasma under the same conditions.

### Self-immolation kinetics

To trigger self-immolation of the linker, a solution of pHLIP(Laa)-STINGa was treated with dithiothreitol (DTT). At different time points (from 30 min to 2 hours of treatment) the samples were analyzed by HPLC using a Zorbax SB-C18 4.6 × 250 mm, 5 μm column with a gradient from 10% to 75% acetonitrile in water containing 0.05% TFA. The chromatograms were recorded at 220 nm, 280 nm and 320 nm. diABZI and pHLIP were used as controls at the same HPLC conditions.

### Biophysics studies

The interactions of pHLIP(Laa)-STINGa with liposomes were investigated by recording the construct’s fluorescence and circular dichroism (CD) using a PC1 spectrofluorometer (ISS) and a MOS-450 spectrometer (Bio-Logic Science Instruments), respectively, with temperature control set to 25°C. Liposomes, constituting of large unilamellar vesicles were prepared by extrusion. 1-palmitoyl-2-oleoyl-*sn*-glycero-3-phosphocholine (POPC) lipids (Avanti Polar Lipids) in chloroform were desolvated on a rotary evaporator and dried under vacuum for a minimum of 2 hours. The phospholipid film was rehydrated in 2 mM citrate phosphate buffer, pH 7.3, vortexed, and passed through the extruder (using a 50 nm membrane pore size) 21 times.

Fluorescence spectra were recorded from 310 nm to 550 nm at an excitation wavelength of 295 nm and 1.0 mm sized slits. The excitation polarizer was set to 54.7 degrees (“magic angle”) while the emission polarizer was set to 0 degrees in order to reduce Wood’s anomalies. CD spectra were recorded from 190 to 260 nm with step size of 1 nm. The concentrations of pHLIP-STINGa and POPC were 7 μM and 1.4 mM, respectively. Also, the fluorescence of STINGa (diABZI) was recorded when excited at the 295 nm and 350 nm wavelengths.

The pH-dependent insertion of pHLIP-STINGa into the lipid bilayer of POPC liposomes was studied by monitoring either the changes in fluorescence intensity at 400 nm or changes in the molar ellipticity at 230 nm as a function of pH. After the addition of aliquots of citric acid, the pHs of solutions containing pHLIP-STINGa and POPC liposomes were measured using an Orion PerHecT ROSS Combination pH Micro Electrode and an Orion Dual Star pH and ISE Benchtop Meter. The normalized fluorescence intensity or millidegree ellipticity values were plotted as a function of pH. The pH-dependence was fit with the Henderson-Hasselbach equation to determine the cooperativity (*n*) and the mid-point *pK* ()f transition. The fitting equations used were


$$pH\;dependence=SII+\frac{SIII-SII}{1+10^{n\left(pH-\text{pK}\right)}}$$

for a single transition and


or two transitions $pH\;dependence=SII+\;\frac{SII^{\prime }-SII}{1+10^{n1\left(pH-\text{pK}1\right)}}+\frac{SIII-SII'}{1+10^{n2\left(pH-\text{pK}2\right)}}$

where *SII* and *SIII* represent spectral signals in state II and III, respectively, and *SII′* represents the CD signal in intermediate between II and III state.

Fluorescence kinetics was measured using a SFM-300 mixing system (Bio-Logic Science Instruments) in combination with the MOS-450 spectrometer with temperature control set to 25°C. All samples were degassed before measurements to minimize air bubbles in the samples. pHLIP-STINGa and POPC samples were incubated overnight to reach equilibrium, when most of the agent was associated with liposome lipid bilayers. To follow pHLIP-STINGa insertion into a membrane, a solution containing 14 μM pHLIP-STINGa and 2.8 mM POPC was mixed with citric acid to lower the pH from pH 8 to 3.5. To monitor fluorescence intensity changes during pHLIP-STINGa insertion into POPC liposomes induced by the pH drop, the emission signal was observed through a cut-off 320 nm filter at an excitation of 295 nm.

For oriented circular dichroism (OCD) measurements, supported bilayers were prepared on quartz slides with special polish for far UV measurements (Starna). The procedure of slide cleaning included the following steps: 1) soaking in cuvette cleaner solution for 24 hours, 2) rinsing with de-ionized distilled water, 3) sonicating for 10 min in 2-propanol, 4) sonicating in acetone, 5) sonicating in 2-propanol once again, 6) rinsing with de-ionized water, 7) soaking in a piranha solution consisting of 25% hydrogen peroxide and 75% sulfuric acid, and 8) rinsing with Milli-Q purified water. A POPC lipid monolayer was deposited on a quartz substrate by the Langmuir-Blodgett (LB) method using (KSV minitrough). For the LB deposition, a small amount of POPC lipid in chloroform was spread on the surface of the subphase and solvent was allowed to evaporate for about 10 min. Next, the monolayer was compressed to 32 mN/m. When the surface pressure was stabilized the first slide was inserted into the trough and held there for 60 seconds so the surface pressure would stabilized again, then it was pulled out from the subphase with speed of 10 mm/min. The second layer was created by fusion with POPC vesicles. About 80 μl of either samples containing 7 μM pHLIP-STINGa and 0.7 mM POPC in 2 mM pH 5.0 or in 2 mM pH 3.3 citrate phosphate buffers or POPC blank containing 0.7 mM POPC (no pHLIP-STINGa) in citrate phosphate buffer was spread onto the slide. The process was repeated for eight more slides, then they were stacked on top of each other. The spacers between the slides kept them from sticking to each other. The “0-hour” OCD spectra were measured for samples at both pHs and POPC blank. Then, slides were kept at 100% humidity at 4°C for 6 hours. After 6 hours, excess solution was shaken off each slide and replaced with 80 μL of buffer of corresponding pH. The slides were again stacked together while filling with the buffer to have a complete set of 8 slides (16 bilayers) and stored at 100% humidity at 4°C for another 6 hours. At the end of the 12-hour incubation period, the “12-hour” OCD spectra were measured. The POPC blank OCD spectrum was subtracted from the OCD spectra of samples.

All data were fit to the appropriate equations by nonlinear least squares curve fitting procedures employing the Levenberg Marquardt algorithm using Origin 8.5.

### Activation of IFN in cells

THP-1-Blue™-ISG cells (Invivogen) expressing an interferon (IFN) regulatory factor (IRF)-inducible secreted embryonic alkaline phosphatase (SEAP) reporter construct were used. Cells were maintained in RPMI growth medium supplemented with L-glutamine, sodium pyruvate, 10% fetal bovine serum (FBS), normocin and ciprofloxacin hydrochloride in a humidified atmosphere of 5% CO_2_ and 95% air at 37°C. Cells were seeded in 96-well plates at a density of 75,000 cells/well. To generate M2 polarized macrophages, cells were treated first with 185 ng/mL phorbol 12-myristate 13-acetate (PMA) for 6 hours and then 20 ng/mL of interleukin 4 (IL-4) IL-4 and 20 ng/mL of interleukin 13 (IL-13) (both from PeproTech) were added for another 16 hours of treatment. At the completion of polarization, the growth medium was replaced with Dulbecco’s Modified Eagle Medium (DMEM) medium without FBS, pH 6.9, containing increasing amounts of pHLIP(Laa)-STINGa or STINGa (up to 10.0 µM). After a two-hour incubation, an equal volume of RPMI supplemented with 20% heat-inactivated FBS was added, and cells were incubated for another 48 hours. SEAP activity was accessed using the QUANTI-Blue™ Solution (Invivogen) to evaluate type I interferon protein levels: 150 μl of the colorimetric reagent was added to 50 μl of cell supernatant for 30 min, 37°C, followed by absorption measurement at 655 nm.

### Cell viability

THP1 cells (ATCC, TIB-202) were maintained in RPMI growth medium supplemented with 2-mercaptoethanol, 10% FBS and ciprofloxacin hydrochloride in a humidified atmosphere of 5% CO_2_ and 95% air at 37°C. Cells were seeded in 96-well plates at a density of 30,000 cells/well. To generate M2 polarized macrophages, cells were treated first with 185 ng/mL PMA for 6 hours alone and then 20 ng/mL of IL-4 and IL-13 were added for another 16 hours of treatment. At the completion of polarization, the medium was replaced with DMEM without FBS, pH 6.4, containing increasing amounts of pHLIP(Laa)-STINGa. After a three-hour incubation, an equal volume of RPMI supplemented with 20% FBS was added. Cell viability was assessed after 48 hours using the CellTiter 96 AQ_ueous_ One Solution Cell Proliferation Assay (Promega); the colorimetric reagent was added to cells for one hour, followed by absorption measurement at 490 nm.

### Treatment of mice

All animal studies (unless it stated differently) were conducted at the University of Rhode Island (URI) according to the approved by URI Institution Animal Care and Use Committee (IACUC) animal protocol AN04-12-011. The studies complied with the principles and procedures outlined by the National Institutes of Health for the care and use of animals.

For the treatment of CT26 tumors, 5x10^4^ CT26 murine colorectal cancer cells (ATCC, CRL-2638) were injected subcutaneously (SQ) in 100 μl of growth medium into the right flank of female Balb/c mice or athymic female nude mice (strain Hsd Athymic Nude-Foxn1nu) ranging in age from 7 to 9 weeks (both from Envigo RMS, Inc). On day 1, when tumors reached size of 100 mm^3^ (“small tumors”) or 400-700 mm^3^ (“large tumors”), mice were randomized into groups, body weight was measured and agents including pHLIP(Laa)-STINGa, pHLIP(Daa)-STINGa, pHLIP(Laa), STINGa (diABZI) or vehicle were given as a single intraperitoneal (IP) or intravenous (IV) injection. Vehicle, pHLIP(Laa), STINGa (diABZI) and pHLIP(Laa)-STINGa were given as a single IP injection of 100 μM 300 µl. pHLIP(Daa)-STINGa was given as a single IV injection of 200 μM 150 μl. The compounds were dissolved in DMSO as a stock solution and transferred to 20% PEG400 in saline containing 0.9% sodium chloride (vehicle). The residual DMSO in the final solution injected into animals was less than 2%. Tumor volume and body weight were measured 3 times per week throughout the study. Measurements of tumors were performed using calipers, and the tumor volume (*V*) was calculated with the formula:


. $$V=0.52\cdot L\cdot W^{2}$$

where *L* is the length and *W* is the width of the measured tumor. Mice were removed from the study and euthanized when the tumor volume was greater than 2000 mm^3^.

Mice in the pHLIP-STINGa treated group, which stayed tumor-free, were re-challenged with tumor cells injected into the opposite flank on day 61 after a single injection of pHLIP-STINGa. Tumor-free mice were kept for additional 40 days (total of 100 days after the treatment with pHLIP-STINGa) and most of them were euthanized. Five tumor-free mice on day 101 received another SQ injection of 10^5^ 4T1 murine breast cancer cells (ATCC, CRL-2539) into their right flanks. Also, a control group of female Balb/c mice received 10^5^ 4T1 cancer cells into the right flank and tumor growth was compared between groups.

For the treatment of 4T1 triple negative breast tumors, 10^5^ 4T1 murine breast cancer cells (ATTC, CRL-2539) were injected SQ in 100 μl of growth medium into the right flank of Balb/c female mice ranging in age from 7 to 9 weeks (Envigo). On day 1, when tumors reached 100 mm^3^ in volume, the body weight was measured, and mice were randomized into four groups. On day 1 mice from groups #2 and #4 received a single IP injection of pHLIP(Laa)-STINGa (100 μM 300 μl). On days 4, 9, 14 mice from groups #3 and #4 received three IP injections of anti-mouse PD-1 antibody (BioCell, CD279), 250 μg/mouse per injection. Mice from the control group (group #1) did not receive any treatment. Tumor volumes and body weight were measured 3 times per week, and mice were euthanized when the tumor volume was greater than 1500 mm^3^.

### ELISA on blood and tumor samples

To establish levels of cytokines in blood and tumor samples, 5x10^4^ CT26 cancer cells were injected SQ in 100 µl of growth medium into the right flank of female Balb/c mice. When tumors reached 150-250 mm^3^ in volume, the mice received a single IV or IP injection pHLIP(Laa)-STINGa or pHLIP(Daa)-STINGa, STINGa or no injections. Animals were euthanized at 4-, 16- and 24-hours post-injection, blood and tumors were collected. Blood samples were kept for 40 min at RT, centrifuged at 5000 g for 20 min at +4°C and supernatant (serum) was collected. Tumors were frozen in liquid nitrogen. Both, the serum and tumor tissue samples were kept at -80°C until further processing and analysis. The tumor samples were processed while on ice, using a bullet blender (Next Advance) with 1 mm diameter zirconium silicate beads (Next Advance). The supernatant of the processed tumors was used for enzyme-linked immunoassay (ELISA) assays. Matched antibody pair kits for mouse tissue necrosis factor alpha (TNF-α) (Sino Biological), mouse interleukin 6 (IL-6) (Abcam), and a pre-coated plate for mouse IFN-β (PBL Assay Science) were used. ELISA assays were performed using the serum and tumor samples. For TNF-α the capture antibody was diluted in phosphate buffer saline (PBS) (Sigma-Aldrich), and for IL-6 the capture antibody was diluted in coating buffer (Abcam). The diluted capture antibodies were incubated in the plates overnight at +4˚C and washed the next day with PBS/Tween washing buffer (Sigma-Aldrich). The TNF-α plate was blocked using 2% bovine serum albumin (BSA) (Thermo Scientific) in washing buffer, while the IL-6 and IFN-β plates are blocked using dilution buffers from the corresponding kit. Blocking was done for 2 hours at RT on an orbital shaker at 200 rpm. After blocking, the plates were washed and then incubated with the diluted tumor and serum samples along with the corresponding standard solutions for each ELISA kit. The samples were incubated for 2 hours at RT on an orbital shaker at 200 rpm. The TNF-α and IFN-β plates were incubated for 1 hour at RT with a diluted detection antibody conjugated with horseradish peroxidase (HRP). The IL-6 plate was incubated for 1 hour at RT with a diluted detection antibody conjugated with biotin followed by incubation for 1 hour at RT with the diluted HRP-streptavidin conjugate (Abcam). All plates were washed and incubated with 3,3’,5,5’-tetramethylbenzidine (TMB) (Invitrogen) and peroxide solution mixed at a ratio of 1:1 (Thermo Scientific) for up to 20 min, then stop solution (10% H_2_SO_4_) was added to the plates. The signal from the wells was quantified by absorbance measured at 450 nm using a Bio-Rad iMark microplate reader. Different dilution schemes were tested in duplicate and antibody standards were used to plot calibration curves.

### Biodistribution, PK and imaging

For pharmacokinetics (PK), biodistribution, pH imaging studies and immunohistochemistry, 5x10^4^ of CT26 cancer cells were injected SQ in 100 µl of growth medium into the right flank of female Balb/c mice and tumors were grown until they reached 150-200 mm^3^ in volume. For PK and biodistribution studies, a single tail vein injection of 200 μM 100 μl of ICG-pHLIP(Laa)-STINGa was performed. Animals were euthanized at 2, 4, 24, 48, 72 and 96 hours post-injection, blood was collected in K_2_ EDTA vacutainer blood collection tubes (BD), and necropsy was performed immediately after euthanization. Blood, tumors and major organs (kidney, liver, spleen, pancreas, lung, heart, large and small intestines, bone, muscle, brain) were collected, and imaged *ex vivo* immediately after necropsy. Blood (150 µl) was imaged in 96-well plate with black bottom and walls. The zero-time point (0 min) was obtained by imaging of ICG-pHLIP-STINGa diluted in blood collected from a control mouse that did not receive any injection (the dilution was made based on the assumption that a mouse contains 80 ml/kg of blood). The fluorescence at zero-time point was taken as 100% and fluorescence recorded at 2, 4, 24 and 48 hrs p.i. were calculated as a percentage of zero-time point signal. The points were fitted using single exponential decay function to establish half-life time.

For *in vivo* imaging, Balb/c and athymic nude mice were given single IP (200 μM 100 μl) or IP (300 μM 150 μl) injections of ICG-pHLIP(Laa)-STINGa when tumors reached 150-250 mm^3^, and *in vivo* imaging was performed at 1, 2, 4, 24, 40-48, 74, 100, 170 and 195 hrs p.i.

For pH imaging studies mice were separated into 2 groups. On day 1, mice from group #1 received a single IP injection of pHLIP(Laa)-STINGa (100 μM 300 µl), while mice from the control group #2 did not receive any treatment. On day 3, mice from groups #1 and 2 received a single IP injection of 50 μM 100 µl of the acidity imaging probe ICG-pHLIP (Iris Biotech, GmbH). On day 4 (or 24 hours after ICG-pHLIP injection) all animals were euthanized, tumors were collected, cut in half and imaged.

The *in vivo* and *ex vivo* bright field and near-infrared fluorescent imaging was performed using a Stryker 1588 AIM endoscopic system with L10 AIM Light Source (808 nm excitation and collection of light in the range of approximately of 815 to 850 nm), and a 1588 AIM Camera using a 10 mm scope. The lens was kept at a fixed distance from the surface of the organs, within an enclosed (light-protected) area. The imaging was performed at three different settings. The digital images of organs were saved in the green channel, transferred into 8-bit files and processed using ImageJ program. A threshold was set from pixel intensity in the range from 1 to 255, leaving out the background with pixel intensity 0. Brightfield images were used to establish the borders of the organs and tumors. The calculated total fluorescence intensity and total area of each organ were used to calculate the mean organ fluorescence.

### FACS analysis

Animal studies on mice were conducted at the Charles River Discovery Service in compliance with the principles and procedures outlined by the National Institutes of Health for the care and use of animals. Uptake of Al647-pHLIP(Laa)-STINGa by tumor cells within CT26 tumors was analyzed by fluorescence activated cell sorting (FACS) analysis. 3x10^5^ CT26 cancer cells were injected SQ in 100 µl of 0% matrigel into the flank of female Balb/c mice (age 8 to 12 weeks). When tumors reached 150-250 mm^3^ in volume mice were separated into two groups. Eight mice from the treated group #1 received a single IP injection of Al647-pHLIP(Laa)-STINGa (300 µM 100 µl), and five mice from the control group #2 received single IP injection of vehicle (1% DMSO in PBS). 24 hours later all animals were euthanized, and tumors were collected for processing. Tumor stroma and immune cells were identified using the following markers:

CD4 T cells: CD45^+^, CD3^+^, CD11b^-^, CD4^+^, CD8^-^
CD8 T cells: CD45^+^, CD3^+^, CD11b^-^, CD4^-^, CD8^+^
Treg: CD45^+^, CD3^+^, CD11b^-^, CD4^+^, CD25^+^, FoxP3^+^
mMDSC: CD45^+^, CD3^-^, CD11b^+^, F4/80^-^, Ly6C^high^, Ly6G^-^
gMDSC: CD45^+^, CD3^-^, CD11b^+^, F4/80^-^, Ly6C^low^, Ly6G^+^
M1: CD45^+^, CD11b^+^, F4/80^+^, CD206^-^
M2: CD45^+^, CD11b^+^, F4/80^+^, CD206^+^
DC: CD45^+^, CD11b^+^, CD11c^+^, MHCII^+^, F4/80^-^
CAF: CD3^-^, CD45^-^, CD140b^+^

The staining was performed for tumor samples, Fluorescence Minus One (FMO) controls and Single Color Controls (SCC) as indicated below:

Tumor: Live/Dead, CD45, CD3, CD4, CD8, CD25, FoxP3, CD11b, F4/80, Ly6C, Ly6G, CD206, CD11c, MHCII, CD140b
Tumor-FMO: CD3, CD25, FoxP3, F4/80, Ly6C, Ly6G, CD206, CD11c, MHCII, CD140b
SCC: Unstained, CD45, CD3, CD4, CD8, CD25, FoxP3, CD11b, F4/80, Ly6C, Ly6G, CD206, CD11c, MHCII, CD140b, Live/Dead

The following anti-mouse antibodies were used: CD45-APC-Fire750 clone 30-F11, CD8a-BV650 clone 53-6.7, CD25-BV605 clone PC61, F4/80-PE-Dazzle-594 clone BM8, Ly-6C-FITC clone HK1.4, Ly-6G-BV785 clone 1A8, CD206-BV421 clone C068C2, CD140b-PE clone APB5, CD11c-BV711 clone N418, I-A/I-E-PE/Cy7 clone M5/114.15.2 from BioLegend; CD3e-BUV496 clone 145-2C11, CD4-BUV395 clone GK1.5; CD11b-BUV737 clone M1/70 from BD Biosciences, FoxP3 PerCP-Cy5.5 clone FJK-16s from Thermo Fisher and Live/Dead Aqua-V500 from Life Technologies. The procedure for tumor processing was the following: tumor samples were dissociated according to the manufacturer’s instructions using the gentleMACS™ protocol “Tumor Dissociation Kit”. Samples were filtered through a 70 µm cell strainer and rinsed twice in PBS/2.5% FBS buffer and total sample volumes were measured. Single cell suspensions were prepared in PBS pH 7.4 at 1x10^7^ cells/mL and placed into individual wells of a 96-well plate and kept on ice. All incubation steps were carried out protected from light. The washing was performed by spinning the plate at 300x g (or 400x g) for 3 minutes and discarding the supernatant. Live/Dead reagent was added to each sample and incubated at 4°C for 15 minutes followed by washing. Fc-block (Mu TruStain FcX/anti-FcγRIV, Biolegend) diluted in Staining Buffer (BD) was added to the samples and incubated for 10 min at 4°C followed by addition of cell surface antibodies diluted in Staining Buffer supplemented with Brilliant Stain Buffer Plus (BD) for 30 min at 4°C and consequent washing. Cells were fixed in FoxP3 Fix/Perm solution and incubated for 30 min at room temperature followed washing. For single color controls, one drop of Ultra Comp Beads (Thermo Fisher) was added to each single-color control well. For Live/Dead controls one drop of ArC Amine Reactive Compensation Bead (Life Technologies) were added followed by addition of each antibody to appropriate well. Isotype control-Al647 clone MOPC-21 (BioLegend) was used for Alexa47 channel, where Al647-pHLIP-STINGa was imaged. The incubation steps were followed by washing steps. The number of cells in the control and treated groups and the cellular uptake of Al647-pHLIP-STINGa was established.

### Immuno-histochemistry and imaging

When the tumors reached 150-250 mm^3^ in volume, mice received a single IP injection of Al647-pHLIP(Laa)-STINGa (300 µM 100 µl). Tumors were cryo-sectioned using a ThermoFisher HM525 NX to make 10-20 µm sections. Sections were stained with fluorescent antibodies, CD206-AL594 (BioLegend), CD68-AL594 (BioLegend), CD140b-AL488 (Invitrogen) and 4′,6-diamidino-2-phenylindole (DAPI) (Sigma-Aldrich) or hematoxylin and eosin (H&E) using hematoxylin 7211 (ThermoFisher) and eosin Y (Poly Scientific). Sections were dried in air for 10 min, then washed with distilled water for 2 min followed by fixation in 4% paraformaldehyde 37% (Sigma-Aldrich) for 12 min, washing with Dulbecco′s phosphate buffered saline (DPBS) (Sigma-Aldrich) for 5 min and drying in air for 10 min. A cover slide was placed on a layer of petroleum jelly (Equate), which was applied to the slide around the tissue. Sections were incubated with blocking buffer containing 5% of 10% BSA (ThermoFisher) for 2 hours at RT followed by washing. Sections were treated with antibody in blocking buffer for 2 hours at RT, followed by washing. A coverslip is mounted on top of the tissue using organo/limonene mount. Imaging of the tissue sections were performed on an EVOS Fl Auto 2 fluorescence inverted microscope using 10x, 20x and 40x objectives in brightfield and fluorescent modes with appropriate filters.

### Statistical analysis

The Kolmogorov-Smirnov two-tailed nonparametric test was used to establish *p-levels*. Log rank (by weighting all time points the same), Breslow method (by weighting all time points by the number of cases at risk at each time point) and Tarone-Ware method (by weighting all time points by the square root of the number of cases at risk at each time point) were used to establish *p-levels* for survival plots.

## Results

### pHLIP-STINGa synthesis and characterization

**Figure 1:**
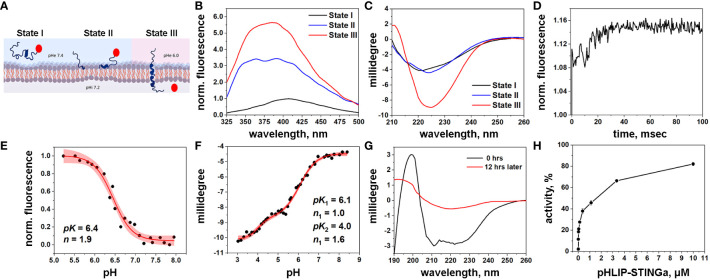
Characterization of pHLIP-STINGa **(A)** Schematic presentation of pHLIP-STINGa interaction with a membrane lipid bilayer (the pHLIP peptide is shown in dark blue and STINGa is shown by a red circle). In state I, pHLIP-STINGa forms an unstructured polymer in solution at normal pH. State II shows the interaction of pHLIP-STINGa with the membrane at normal pH. State III represents the transmembrane helical orientation of pHLIP triggered by low pH, which leads to the translocation of STINGa across the lipid bilayer and its release in the cytoplasm. The three states were monitored by changes of fluorescence **(B)** and CD **(C)** spectral signals of pHLIP-STINGa interacting with POPC liposomes. **(D)** Kinetics of fluorescence changes triggered by pH drop in presence of POPC liposomes is shown. pH transitions monitored by changes of fluorescence intensity **(E)** and CD **(F)** spectral signals are shown (experimental points and fitting curves, red, with 95% confidence interval, pink). **(G)** OCD spectra of pHLIP-STINGa recorded immediately after deposition of pHLIP-STINGa on the supported bilayer and 12 hrs later, when the insertion of pHLIP-STINGa into bilayer was complete, are shown. **(H)** Activation of the IFN signaling pathway induced by pHLIP-STINGa in THP1-Blue-ISG cells polarized by PMA, IL-4/IL-13 into M2-type macrophages is shown. The results were normalized to the activity of STINGa alone at the maximum concentration tested, which was taken as 100%.

pHLIP peptides sense cell surface acidity and insert across the plasma membranes of acidic cells. Our approach is to attach a STINGa molecule to the inserting end of a pHLIP, by using a self-immolating linker to release the agonist in the cytoplasm (**Figure 1A**). We synthesized pHLIP-STINGa using dimeric diABZI (11), which was conjugated *via* a self-immolating disulfide cleavable linker to the membrane-inserting end of pHLIP peptides consisting either of all L or all D amino acids (Laa and Daa) (**Figure S1**). Both pHLIP(Laa)-STINGa and pHLIP(Daa)-STINGa were used in the mouse experiments. In stability studies, we found that about 40% of modified STINGa is released from pHLIPs when either the Laa or Daa agents are incubated with mouse plasma (Supplementary Information **Table S1**); however, the stability was significantly higher for pHLIP(Daa)-STINGa in human plasma, where only ~10% degradation is observed over 24 hours. Also, pHLIP(Daa)-STINGa exhibited much stronger binding to plasma proteins than the Laa version, especially to human plasma proteins.

pHLIP is expected to translocate STINGa across the membrane into the cytoplasm, where the S-S bond will be reduced, followed by linker self-immolation and release of the original, unmodified STINGa as a dimeric diABZI. In a model experiment we induced cleavage of the S-S bond by dithiothreitol (DTT) and observed that 88% of immolation was completed within 1 hour, releasing STINGa in its unmodified form (**Figure S2**).

### Biophysical characterization of pHLIP-STINGa interactions with a membrane

Biophysical studies using POPC model liposomes confirmed the pH-dependent interactions of pHLIP-STINGa with the membrane lipid bilayer. The fluorescence of pHLIP-STINGa excited at 295 nm exhibits pH-dependent behavior (**Figure 1B**). However, the fluorescence spectra are shifted to longer wavelengths compared to typical tryptophan emission and double peaks are observable. This behavior occurs since diABZI emits light in the range of 350-500 nm when it is excited at 295 nm (**Figure S3**) contributing to the emission of the tryptophan residues of the pHLIP peptide. Energy transfer from tryptophan (Trp) residues of pHLIP to diABZI occurs, since the diABZI excited at 322 nm exhibits even stronger fluorescence (**Figure S3**) contributing to the overall pHLIP-STINGa emission. Circular dichroism (CD) measurements show the expected formation of helical structure at low pH as the peptide inserts across the bilayer (**Figure 1C**). Kinetics studies of pH-triggered bilayer interactions of pHLIP-STINGa revealed fast insertion (complete in 30 msec) (**Figure 1D**). A pH-dependence graph of changes of fluorescence intensity during the transition from the membrane-bound to the membrane-inserted pHLIP-STINGa conformation revealed that the transition occurs with a pK of 6.4 (**Figure 1E**). The pH-dependence graph of changes of CD signal revealed 2 transitions, one with a pK of 6.1 and another with a pK of 4.0 (**Figure 1F**). To confirm that the pK 6.1 transition is to a transmembrane orientation of pHLIP-STINGa, we recorded oriented CD (OCD) spectra at different pH values, both immediately after deposition of solution of pHLIP-STINGa and POPC in supported layers and 12 hours later, when the insertion process is complete. The results show that the agent inserts into the lipid bilayer and adopts a transmembrane orientation at a pH around 5 (**Figure 1G**). The OCD spectra recorded at pH 5 and pH 3.3 are shown in **Figure S4**. The second transition might be from final conformational adjustments of pHLIP-STINGa in the membrane after its insertion and the translocation of the C-terminal end of pHLIP linked to diABZI.

To complete the mechanistic characterization of pHLIP-STINGa, activation of the interferon signaling pathway was confirmed in activated macrophages. Activation was studied in THP1-Blue cells derived from the human THP-1 monocyte cell line by stable integration of an interferon regulatory factor (IRF)-inducible secreted embryonic alkaline phosphatase (SEAP) reporter construct. We found that THP1-Blue cells exhibited a concentration-dependent activation of IRF signaling when polarized by phorbol 12-myristate 13-acetate (PMA), interleukin 4 (IL-4) and interleukin 13 (IL-13) into M2-type macrophages and treated with pHLIP-STINGa. As shown below, M2-type macrophages are the most abundant immune cells within the CT26 tumors we investigated. Thus, we confirmed successful intracellular delivery and release of STINGa by pHLIP (**Figure 1H**). pHLIP-STINGa did not induce death of these cells as confirmed by a cytotoxicity assay (**Figure S5**).

### Biodistribution and pharmacokinetics

**Figure 2:**
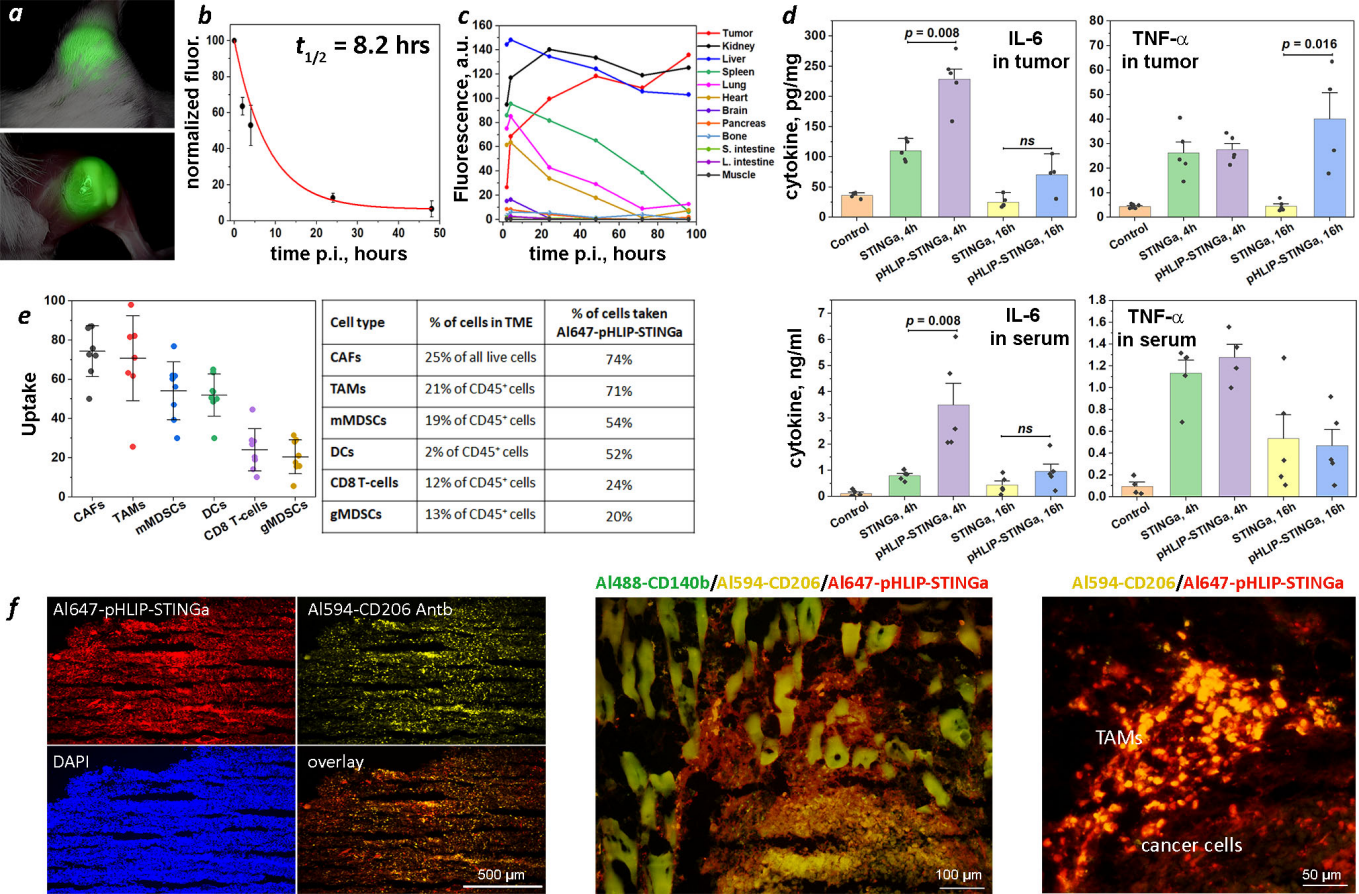
Tumor targeting, PK, biodistribution, tumor and serum cytokines, tumor stroma and immune cell uptake. **(A)** Imaging of CT26 tumor targeting in mouse performed at 40 hrs after single IP injection of ICG-pHLIP-STINGa (100 μM 300 μl). Tumor site prior to and after removal of skin is shown. **(B)** Normalized fluorescence recorded in blood, which was collected at different time points after single IV injection of ICG-pHLIP-STINGa (200 μM 100 μl), is shown (mean and SE). The data were fitted by exponential function (red line). **(C)** Kinetics of ICG-pHLIP-STINGa targeting of CT26 tumor and clearance of the agent from major organs are shown. The mean fluorescence per area was calculated for each organ and tissue collected at different timepoints after single IV injections of ICG-pHLIP-STINGa (200 μM 100 μl). **(D)** Level of IL-6 and TNF-α cytokines in tumors and serum established by ELISA at different time points after a single IV injection of STINGa or pHLIP-STINGa (200 μM 150 μl) in comparison to control mice are shown (all points, mean and SE are shown, *p*-levels were calculated using the Kolmogorov-Smirnov two-tailed nonparametric test). **(E)** Percent of populations of tumor stroma and immune cells within TME targeted by Al647-pHLIP-STINGa was established by FACS analysis on CT26 tumors collected 24 hrs after a single IP injection of Al647-pHLIP-STINGa (100 μM 300 μl) (all points, mean, St.d. are shown on graphs and numbers are given in the table). **(F)** Co-localization of Al647-pHLIP-STINGa with Al488-CD140b-Antb staining CAFs, Al594-CD206-Antb staining TAMs and DAPI staining cell nuclei within TME are shown on images obtained at different magnifications using 10x, 20x and 40x objectives. ns means non-significant.

For pharmacokinetics (PK), biodistribution, tumor uptake and immuno-histochemistry studies, fluorescent dyes, either near infrared indocyanine green (ICG) or Alexa647 (Al647), were conjugated to the non-inserting end of the pHLIP to make ICG-pHLIP-STINGa or Al647-pHLIP-STINGa fluorescent agents. ICG-pHLIP-STINGa was given as a single intravenous (IV) injection when CT26 tumors established in the right flanks of female Balb/c mice reached volumes of about 150-300 mm^3^. The murine CT26 colon tumor cell line is a well-established model for testing immuno-oncology therapeutics and it exhibits a weak response to ICIs. Fluorescence imaging *in vivo* shows tumor targeting (**Figure 2A**), and the fluorescence signal in the tumor persists for 8 days. For biodistribution and PK studies, the animals were euthanized, followed by necropsy, blood, organs and tissue harvesting and imaging at 2-, 4-, 24-, 48-, 72- and 96-hour time points after a single IV injection of ICG-pHLIP-STINGa. Whole blood imaging was used to establish clearance of the agent (**Figure 2B**). The half-life of ICG-pHLIP-STINGa is 8.2 hours, which is ~6x higher compared to the 1.4 hours of half-life of STINGa (diABZI) on its own (11). Thus, pHLIP improves the PK and extends the circulation time of STINGa in the blood. The calculated mean fluorescence per area measures the uptake of ICG-pHLIP-STINGa by the organs (**Figure S6**). The kinetics of the fluorescence signal of ICG-pHLIP-STINGa indicate a significant accumulation of the agent in the tumor in 48 hours followed by a continuing slow increase of the signal up to 100 hours (**Figure 2C**). The liver signal decreases slowly, suggesting some hepatic clearance, which might arise from the presence of the ICG dye (known to be predominantly cleared by the liver). The kidney signal also increases initially, possibly indicating some renal clearance of the agent as well, with a slow decrease at later time points. Animals were euthanized without flushing with buffered saline, meaning the organs were imaged with blood in them, so the measurement would include any agent remaining in the organ blood at a given time point. The signal in the spleen, heart and lungs peaked at 4 hours (reflecting slow blood clearance of the agent) followed by a decay. The fluorescence in the brain, bone, pancreas, muscle, and small and large intestines was low or undetectable. We note that the presence of the agent in an organ does not mean cellular delivery of STINGa, which occurs in a low pH environment only causing pHLIP to insert into cellular membranes, followed by STINGa cleavage from pHLIP, linker self-immolation, and release of STINGa.

### pHLIP targets STINGa to TAMs, CAFs, mMDSCs and DCs and activates cytokines

Cytokine levels induced in tumors and serum were measured following a single IV injection (200 µM 150 μl) of STINGa or pHLIP-STINGa (**Figure 2D**). The levels of IL-6 in tumors at 4 hrs p.i. and TNF-α in tumors at 16 hrs p.i. were 2.1 and 8.9 times higher respectively, after administration of pHLIP-STINGa compared to STINGa. To directly evaluate the amounts of IL-6, TNF-α and IFN-β in tumor and serum after a single injection of pHLIP-STINGa, the data are given in ng of cytokine per ml of tumor supernatant or serum in **Figure S7**. The amounts of IL-6 and TNF-α in tumor supernatants were 3.5-7.5 times and 5-6 times higher, respectively, compared to the corresponding levels of these cytokines in the blood. A transient increase of IFN-β was seen in the blood 4 hrs p.i., however within the next 12 hrs the level of this cytokine dropped to zero. The data show an enhanced level of cytokines generated within tumors after a single administration of pHLIP-STINGa.

pHLIP targeting of highly proliferative and metabolically active cancer cells with low cell surface pH has been demonstrated numerous times (12–15). Recent data shows that tumor stroma and activated immune cells with TME are also targeted by pHLIP agents (16, 17). This additional targeting is important for therapy, since the CAFs forming tumor stroma constitute about 25% of all live cells within the TME, and M2-type TAMs and mMDSCs representing activated myeloid cells within the TME of CT26 tumors collectively constitute about 40% of all immune (CD45^+^) cells and 15% of all live cells. We performed a fluorescence activated cell sorting (FACS) analysis of the cells from CT26 tumors in mice 24 hrs after a single intraperitoneal (IP) injection of Al647-pHLIP-STINGa (300 µM 100 μl). We established that 74% of all CAFs (CD3^-^, CD45^-^, CD140b^+^) and 71% of CD206^+^ TAMs (CD45^+^, CD11b^+^, F4/80^+^, CD206^+^) were targeted by Al647-pHLIP-STINGa (**Figure 2E**). Also, about 50% of mMDSCs (CD45^+^, CD3^-^, CD11b^+^, F4/80^-^, Ly6C^hi^, Ly6G^-^) and DCs (CD45^+^, CD11b^+^, CD11c^+^, MHCII^+^, F4/80^-^) were targeted by the agent, and about 20% of CD8 T-cells (CD45^+^, CD11b^-^, CD3^+^, CD4^-^, CD8^+^) and gMDSCs (CD45^+^, CD3, CD11b^+^, F4/80^-^, Ly6C^lo^, Ly6G^+^) were targeted by Al647-pHLIP-STINGa. DCs constitute 2% of all immune (CD45^+^) cells and 0.7% of all live cells; gMSDCs constitute 13% of all immune (CD45^+^) cells and 5% of all live cells, and CD8 T-cells constitute 12% of all immune (CD45^+^) cells and 4% of all live cells within CT26 tumors. Targeting of the most abundant non-cancer cells within TME, CAFs and CD206^+^ macrophages, was confirmed by immunohistochemistry performed on CT26 tumors collected 24 hrs after Al647-pHLIP-STINGa administration (**Figure 2F**). Thus, it is expected that each of the metabolically hyperactive cell types in the tumor, including the stroma, will receive the STING agonist delivered by pHLIP.

### Eradication or tumors and development of immunity

**Figure 3:**
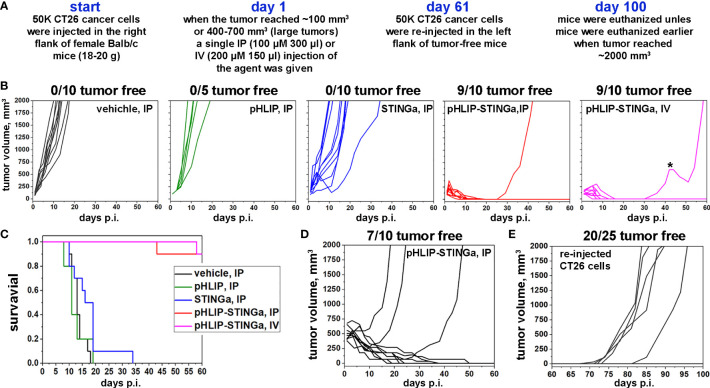
Eradication of CT26 tumors and development of immune memory. **(A)** Experimental design. **(B)** CT26 tumor growth curves in Balb/c mice are shown after a single administration of different agents at dose levels of 100 μM 300 μl for IP injections or 200 μM 150 μl for IV injections performed on day 1, when tumors had reached about 100 mm^3^ in volume. The star (*) indicates that this mouse in IV group received a second dose of pHLIP-STINGa on day 41, when the tumor had started to re-grow. **(C)** Kaplan-Meier survival plots obtained for the data shown in panel **(B)**. **(D)** CT26 large tumor growth curves are shown after a single IP injection of pHLIP-STINGa (100 μM 300 μl) on day 1, when tumors had reached 400-700 mm^3^ in volume. **(E)** CT26 tumor growth curves obtained for tumor-free mice from the groups that received single IP or IV injections, small and large tumors, of pHLIP-STINGa on day 1. On day 61 CT26 cancer cells were re-injected in the left flanks of these mice and tumor growth was monitored for additional 40 days.

The experimental design of therapeutic studies is shown in **Figure 3A**. CT26 cancer cells were inoculated into the right flank of Balb/c mice. When a tumor reached about 100 mm^3^ in volume, designated as day 1, a single IP (100 µM 300 μl) or IV (200 µM 150 μl) injection was administered to mice assigned in four groups: vehicle (20% PEG400/0.9% NaCl); pHLIP; STINGa; or pHLIP-STINGa, where pHLIP(Laa)-STINGa was used in the IP injection group and pHLIP(Daa)-STINGa was used in the IV injection group. Tumor growth was monitored for 60 days or until the tumor volume endpoint was achieved (2000 mm^3^, when animals were euthanized) (**Figure 3B**). All animals in the control groups that received either vehicle or pHLIP developed tumors within 18-20 days p.i. In the group that received STINGa at the same molar dose level as pHLIP-STINGa tumor growth was slightly delayed, however by day 35 p.i. all mice had developed tumors. In the experimental group that received pHLIP-STINGa, consisting of the IP (10 mice) and IV (10 mice) groups, the tumors disappeared, and 18 out of 20 mice remained tumor free for 60 days after the administration of the agent. One mouse in the IV group received another dose of pHLIP-STINGa on day 41, when the tumor started to re-grow, but it did not alter the outcome, the tumor continued to grow. The Kaplan-Meier survival plots demonstrate dramatic differences between the control and STINGa groups versus the pHLIP-STINGa groups (**Figure 3C**).

Larger tumors were also studied, since CT26 tumors of different sizes have different immune cell compositions (18). The proportion of all immune cells drops from 65% of all live cells in small tumors (~100 mm^3^) to 20% in large tumors (~500 mm^3^), and the population of CD3^+^ cells drops from 28% to 7%. Larger tumors contain more CD11b^+^ myeloid cells. The proportion of immune cells and their composition typically affects treatment efficacy. To study the treatment of larger tumors, a single IP injection of pHLIP-STINGa (100 µM 300 μl) was given when CT26 tumors in Balb/c mice reached a volume of 400-700 mm^3^. Three out of 10 mice developed larger tumors by 60 days, while the other mice slowly fought the cancer and became tumor-free (**Figure 3D**). It took about 20-30 days for the tumors to be eradicated in these mice.

To examine the development of immunity the 25 mice from the pHLIP-STINGa IP/IV groups (9 mice from IP small tumor, 9 mice from IV small tumor and 7 mice from IP large tumor groups) that had remained tumor-free for 60 days were inoculated with CT26 cells in their previously uninoculated left flanks and were monitored for an additional 40 days. One mouse from IP small tumor group, 2 mice from IV small tumor group and 2 mice from IP large tumor group developed tumors. Thus, 20 out of the 25 mice (80%) remained tumor-free, indicating the development of immune memory and the consequent rejection of the CT26 cancer cells (**Figure 3E**). In a separate experiment, 4T1 triple negative murine breast cancer cells were inoculated into 5 of the mice that had stayed tumor-free for 100 days and had the re-injection of CT26 cells (**Figure S8**). Each of these mice developed 4T1 tumors, indicating that immune memory was developed for CT26 cancer cells, but not for 4T1 cancer cells.

To confirm the importance of T-cells in the process of tumor eradication induced by pHLIP-STINGa, CT26 cancer cells were inoculated into athymic nude mice lacking T-cells. Some delay in tumor growth was observed (**Figure S9**), however survival analysis did not reveal any statistically significant differences between control and treated groups, clearly indicating the importance of T-cells.

We also treated aggressive triple negative 4T1 breast tumors with a single IP injection of pHLIP-STINGa (100 µM 300 μl) when tumors reached a volume of ~100 mm^3^ (designated as day 1) or 3 IP injections on days 4, 9 and 14 of an anti-PD-1 antibody (250 µg/injection) or a combination of pHLIP-STINGa with the PD-1 antibody series. While each treatment delayed the growth of 4T1 tumors slightly (*p*< 0.02), the longest survival was observed for the combination treatment of a single pHLIP-STINGa followed by multiple PD-1 antibody injections (*p*< 0.004) (**Figure S10**). However, in contrast to CT26 tumors, it was not possible to eradicate the 4T1 tumors.

In all treatment groups, the single injection of pHLIP-STINGa (IP or IV) led to transient distress and slight transient weight loss (≤10%), with complete recovery within 3-4 days (**Figure S11**).

### Obliteration of tumor stroma and increase of pH within TME

**Figure 4:**
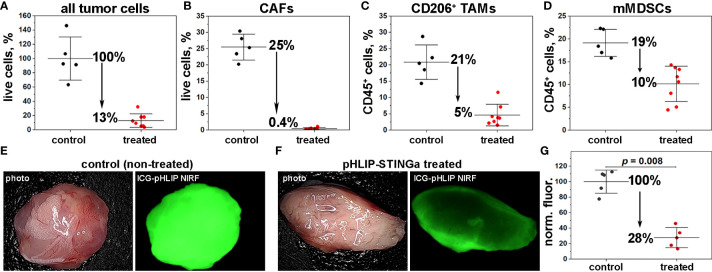
Obliteration of tumor stroma and pH increase within the TME. **(A–D)** The numbers of cells within the TME in control (mice receiving vehicle as a single IP injection) and treated (mice receiving a single IP injection of Al647-pHLIP-STINGa (100 μM 300 μl)) were established by FACS analysis. The percentages of all live cells with TME **(A)**, the number of CAFs (CD3^-^, CD45^-^, CD140b^+^) quantified as % of all live cells **(B)**, the numbers of TAMs (CD45^+^, CD11b^+^, F4/80^+^, CD206^+^) **(C)** and mMDSCs (CD45^+^, CD3^-^, CD11b^+^, F4/80^-^, Ly6C^hi^, Ly6G^-^) **(D)** both quantified as % of CD45^+^ immune cells are shown (all points, mean and St.D., the *p-levels* for all graphs < 0.002). **(E–G)** pH imaging with ICG-pHLIP and quantification of the signal are shown. Mice bearing CT26 tumor in right flank (about 100 mm^3^) were divided into 2 groups (5 animals per group). One group (treated) was treated with a single IP injection of pHLIP-STINGa (100 μM 300 μl) on day 1 and another group (control) did not receive any treatment. On day 3 an ICG-pHLIP pH-imaging probe was given in single IP injections (50 μM 100 μl) to both control (non-treated) and pHLIP-STINGa treated groups, and 24 hrs later (on day 4), animals were euthanized, tumors were collected, cut in half and imaged, the representative images are shown on panels **(E, F)** and normalized mean fluorescence signal calculated for all tumor pieces (5 animals per group) are shown on panel **(G)** (all points, mean and St.D. are shown, *p*-*level* was calculated using the Kolmogorov-Smirnov two-tailed nonparametric test).

To gain a better understanding of the therapeutic efficacy observed for CT26 tumors after a single pHLIP-STINGa administration, we quantified the number of cells in the TME by FACS analysis. Only 13% of the cells remained alive in the tumors at 24 hrs after pHLIP-STINGa treatment (**Figure 4A**). The most dramatic effect was observed for tumor stroma: the amount of CAFs, which constitute 25% of all live cells within CT26 tumors, dropped to 0.4% of all cells within 24 hrs after pHLIP-STINGa administration (**Figure 4B**). The viable CD206^+^ TAMs and mMDSCs dropped 4 and 2 fold, respectively, after the treatment (**Figures 4C, D**). The populations of CD8 T-cells and DCs within TME were reduced after pHLIP-STINGa treatment, while the population of gMDSCs increased from 5% of all live cells in non-treated control to 9% in the treated group (Fig S12).

The significant increase of TNF-α within the TME and the complete destruction of the tumor stroma within 16-24 hrs after pHLIP-STINGa treatment correlates with the enhanced blood flow to tumors and the intra-tumoral hemorrhage observed both in Balb/c and nude mice (**Figure S13**) and potentially with an alteration of tumor pH. Therefore, we investigated how the pH within the TME changes after pHLIP-STINGa treatment. When CT26 tumors reached a volume of about 100 mm^3^, a single IP injection of pHLIP-STINGa (100 µM 300 μl) was given to mice in the “treated” group. On day 3, a single IP injection of the acidity probe, ICG-pHLIP (50 µM 100 μl), was given to the “treated” and “control (untreated)” groups of mice, and 24 hrs later (on day 4), mice were euthanized, tumors were collected, cut in half and imaged (**Figures 4E, F**). Quantification of the signal indicates that the mean ICG-pHLIP fluorescence calculated per area of tumor tissue specimens cut in half in the treated group dropped by 72% compared to the control (untreated) group (**Figure 4G**). Since the level of targeting by ICG-pHLIP reflects the level of tissue acidity, the significant decrease in retention of ICG-pHLIP within treated tumors, especially when the blood flow to tumors is enhanced, shows an increase of pH within acidic CT26 tumors after treatment with pHLIP-STINGa, potentially due to the death (including metabolic death) of tumor stroma and cancer cells and the consequent lack of acid production, which should help enable T-cell and NK-cell activity.

## Discussion

STING agonists are promising but flawed as an approach to using the immune system to fight “cold”, uninflamed tumors. By targeting the STINGa’s to the acidic cells in these tumors, their performance might be enhanced, allowing their use in a larger number of cases. We report a promising approach for targeting active cells within the tumor microenvironment by using a pHLIP peptide and the effective intracellular delivery of STINGa to trigger the destruction of the tumors.

Highly proliferative cancer cells, activated immune cells, and CAFs within the TME tend to switch to glycolysis to rapidly produce energy either in the presence of oxygen (Warburg effect) or in hypoxic conditions (Pasteur effect) (19, 20). Lactate and protons, byproducts of glycolysis, are actively transported from the cytoplasm to the extracellular space (21, 22). Also, cancer cells located next to the stroma (consisting of CAFs and TAMs) can consume lactate and other metabolites promoting the oxidative phosphorylation (OXPHOS) pathway, a phenomenon known as the Reverse Warburg effect (23), establishing a “crosstalk” with stromal cells, which leads to a well-orchestrated proliferation and expansion of tumors. The main byproduct of OXPHOS is carbon dioxide, which can freely diffuse across the membrane along its concentration gradient. CO_2_ is converted to protons and bicarbonate ions (a reaction catalyzed by carbonic anhydrase IX (CAIX) overexpressed on tumor cell surfaces), contributing to the acidification of the extracellular space (24). Thus, either in overactivated glycolysis or in OXPHOS, an excess of protons is generated around metabolically active cells independent of the level of glucose consumption or lactate production. The flux of exported acidity lowers the pH surrounding a tumor cell, and the proton concentration is accentuated near the cell surface both by the flux and by the membrane electrochemical potential. As a result, the extracellular pH is lowest at the surfaces of metabolically active cells, where it is significantly lower than the bulk extracellular pH (25, 26).

pHLIP peptides have been shown to sense cell surface pH and target acidic tumors. At the low cell surface pH of a metabolically overactive cell, several carboxyl groups within a pHLIP become protonated, triggering peptide folding and insertion across the cell membrane to form a stable transmembrane helix. The dielectric environment at the membrane surface shifts the pKa’s of the carboxyl groups toward higher pHs, and a moderately low local pH promotes their protonation. A variety of imaging and therapeutic agents have been successfully delivered to tumors by pHLIP peptides (16, 27–34). Tumor targeting by pHLIP peptides has been shown to be positively correlated with tumor extracellular pH (35, 36) and is enhanced by acidification produced by co-injection of glucose (37) or the overexpression of CAIX (36). Conversely, tumor targeting has been shown to be reduced by the alkalization of tumors in mice fed with bicarbonate drinking water (38). The pHLIP technology has been translated to ongoing clinical trials for PET imaging, fluorescence-guided surgery with ICG-pHLIP and tumor treatment with pHLIP-exatecan (CBX-12).

We used STINGa (diABZI), conjugated to the membrane-inserting end of a pHLIP peptide *via* a S-S cleavable self-immolating linker and found it to have desirable properties. The pK of insertion into a model membrane is 6.1-6.4, which is in the range of pH at the surface of metabolically active cells in diseased tissues. We also found a fast (msec) rate of insertion, well suited for cell targeting *in vivo*. Characterization of the pHLIP-STINGa agent revealed significant binding to plasma proteins. The highest stability and strongest plasma protein affinity was found for pHLIP(Daa)-STINGa (wherein the pHLIP is made from D-amino acids). The half-life of fluorescent pHLIP(Laa)-STINGa in a mouse was established to be about 6 times higher compared to STINGa alone, a significantly improved PK. Slow blood clearance of other pHLIP imaging and therapeutic agents in animals has previously been observed (12, 39, 40) and confirmed in humans. Tumor targeting was confirmed by imaging. Biodistribution clearly indicated the accumulation of the agent in tumors, which continued up to 100 hours post-injection. FACS analysis performed on tumor tissue revealed that more than 50% of all CAFs, M2 TAMs, mMDSCs and DCs are targeted by fluorescent pHLIP-STINGa, and that 20-24% of CD8 T-cells and gMDSCs are targeted as well.

The progression of immune-excluded “cold” tumors is associated with the formation of dense stroma consisting of acidic TAMs and CAFs, generating immuno-suppressive signals and impairing the homing of T-cells and their cytotoxic function (41–44). Targeting of STINGa by pHLIP to CAFs, TAMs, mMDSCs and DCs, and the resulting activation of cytokines (enhanced levels of IL-6 and TNF-α were observed within the TME) led to dramatic therapeutic results. A single IP or IV injection of pHLIP-STING into mice bearing CT26 tumors (~100 mm^3^) triggers complete tumor eradication (90% incidence rate) and the mice remained tumor-free for 60 days after pHLIP-STINGa administration. All mice receiving the same dose of untargeted STINGa developed tumors within 20-35 days. Only 13% of tumor cells remained viable 16-24 h after pHLIP-STINGa administration. A significant increase in the level of TNF-α was observed within the TME. TNF-α triggers the disruption of tumor vasculature, reduces intra-tumoral interstitial fluid pressure (IFP) and promotes the flow of blood to tumors, and TNF-α is used to improve drug delivery to tumors (45). After a single pHLIP-STINGa injection the tumor stroma was severely disrupted. The number of CAFs, which constitute 25% of all tumor cells, was reduced by 98%, and intratumoral hemorrhage was observed. We also observed about an 80% reduction in the population of cytotoxic CD8 T-cells with the TME (their amount dropped from 4.2% of all live cells to 0.9%) within 24 hrs after treatment. Previous reporting is that activation of STING can lead to T-cell apoptosis (46). At the same time, cytokine signaling, destruction of tumor stroma, and reduction of IFP are expected to lead to the recruitment of new T- and NK-cells and to facilitate their free penetration into the tumor mass. Our data are in line with previous findings indicating STING-induced tumor vascular remodeling, which promotes vascular normalization and correlates with enhanced T-cell infiltration and prolonged survival in human colon and breast cancer (47, 48). We also assessed the level of acidity within treated tumors using ICG-pHLIP as an acidity probe: the tumor acidity was significantly reduced (by 72%) within three days after treatment compared to the control group. Thus, not only did T-cells have access to the tumor core, but the T-cell cytotoxic activity unimpaired by an acidic environment.

When tumor bearing mice that became and remained tumor-free after a single injection of pHLIP-STINGa on day 1 were inoculated with CT26 cells on day 61, 15 out of 18 animals (83%) remained tumor free for another 40 days (100 days in total after a single IP or IV injection of pHLIP-STINGa). Thus, a single pHLIP-STINGa injection promoted the development of T-cell immune memory. The importance of T-cells was confirmed in a study performed on athymic nude mice lacking T-cells. pHLIP-STINGa did not exhibit any statistically significant therapeutic efficacy on CT26 tumors in nude mice, while intratumoral hemorrhage was observed as well. We expect that the tumor stroma was destroyed, but the lack of T-cells did not allow tumor eradication.

In addition to the treatment of small (~100 mm^3^) tumors we treated mice with large (~500 mm^3^) CT26 tumors. A single IP injection of pHLIP-STINGa into mice with large tumors (400-700 mm^3^) led to tumor eradication in 7 out of 10 treated animals, and the complete treatment response took 20-40 days. Large tumors have many cancer cells to target [80% of all tumor cells are cancer cells vs 35% of cancer cells in small CT26 tumors (18)], and large CT26 tumors also have a higher population of myeloid cells among all immune cells within TME (87% of CD45^+^ cells). Successful treatment of large CT26 tumors holds promise for the eradication of immunosuppressive tumors with a high number of myeloid cells.

As an extreme case, we also treated aggressive 4T1 triple negative breast tumors in Balb/c mice. The treatment was less successful compared to the treatment of CT26 tumors. A moderate delay of tumor growth was achieved by combining a single injection of pHLIP-STINGa with multiple injections of a PD-1 ICI. 4T1 tumors are similar to large CT26 tumors in the number and composition of immune cells (18). A 4T1 tumor contains a smaller population of all immune cells (31% of all tumor cells) and a high population of CD11b^+^ myeloid cells (75% of CD45^+^ cells). Also, like CT26 tumors, 4T1 tumors are acidic and very well targeted by pHLIP (12, 14, 39). However, a CT26 tumor has 3,023 single nucleotide variations (SNVs) and 362 short indels, while 4T1 tumors have a lower mutational burden, with 505 SNVs and 20 short indel (49). The low tumor mutational burden (TMB) results in a smaller number of neo-antigens at the surface of 4T1 cells and makes 4T1 tumors less immunogenic and less responsive to therapies based on T-cell action, including ICI therapies.

ICIs, which promote the cytotoxic action of T-cells, have dramatically changed the lives of some cancer patients, with unprecedented durable responses and improved survival; however, most patients do not benefit, with response rates ranging from 20-40% (50). Preclinical and clinical evidence suggests that ICIs/T-cell based therapies do not work in “cold” acidic tumors with impaired MHC-I (major histocompatibility complex class I) presentation and low TMB (51–56), since i) T-cells should be present within TME, ii) their cytotoxic function should not be impaired by an acidic environment or signaling; iii) for T-cells to recognize cancer cells and, especially, to develop immune memory, a sufficient number of neo-antigens should be properly presented on cancer cells.

**Figure 5:**
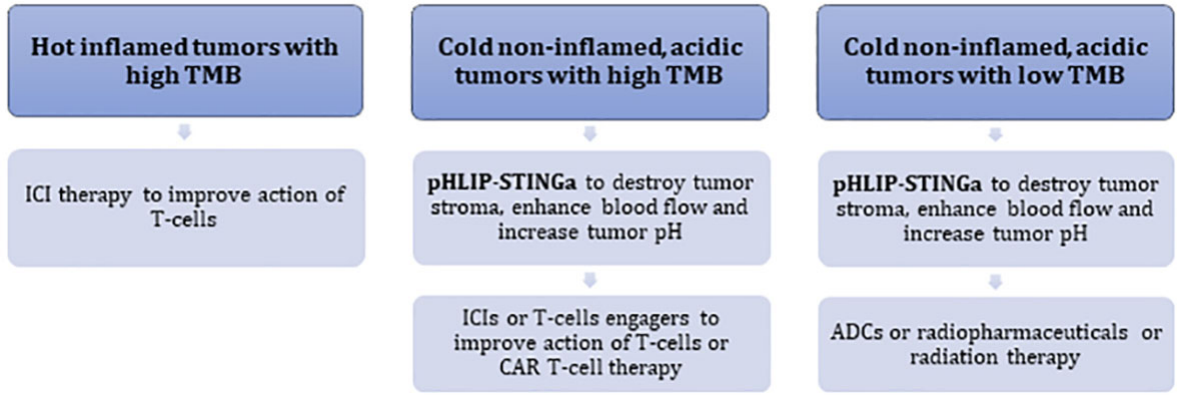
Schematic presentation of different types of tumor development and treatment options.

pHLIP-STINGa can convert “cold”, non-inflamed, T-cell excluding tumors into “hot” tumors with high levels of inflammatory cytokines within the TME, obliteration of the tumor stroma, significant enhancement of blood flow to the tumor core and an increase of tumor pH. In the clinic, acidic “cold” tumors might be identified by a ^89^Zr-pHLIP PET agent, which is currently in translation to clinical trials, and TMB and MHC-I status can be established by genomic analysis. Thus, it may prove possible to identify patients who will not respond to ICIs, and potentially could be treated with pHLIP-STINGa either as a monotherapy or in combination with other therapeutics. pHLIP-STINGa could be combined with ICIs, T-cell engagers, or CAR T-cell therapies to promote access of these therapeutics to the tumor and to enhance their action by normalizing pH conditions. If the TMB is low and MHC-I is lacking, therapies based on the action of T-cells are expected to be less effective. For such tumors, pHLIP-STINGa can destroy tumor stroma, enhance blood flow to tumors and reduce hypoxia. Then, treatments could be continued with antibody-drug conjugates (ADCs), or antibodies labeled with radioactive isotopes, or radiation therapy, which could be significantly enhanced under non-hypoxic conditions. Combinations with pHLIP-STINGa might decrease the effective doses of these therapeutics. Different types of tumor development and potential treatment options are presented in the following scheme (**Figure 5**).

The pHLIP technology may allow transformation of immuno-activating agents into more potent therapeutics, since pHLIP can target and deliver these agents to cancer cells, tumor stroma and myeloid cells. As opposed to delivery targeted to specific receptors on the surfaces of particular cells, pHLIP offers targeting of all (or a majority) of metabolically active cells within the tumor microenvironment. Since the tumor microenvironment is complex, such an approach in targeting and delivery leads to a significant synergistic effect. A single injection of pHLIP with immuno-activator (STINGa) induces production of cytokines, obliterates tumor stroma and increases the tumor pH, which results in the eradication of tumors and the development of immune memory.

## Data availability statement

The raw data supporting the conclusions of this article will be made available by the authors, without undue reservation.

## Ethics statement

The animal study was reviewed and approved by The University of Rhode Island Institution Animal Care and Use Committee, Animal protocol AN04-12-011.

## Author contributions

AM, DE, OA and YR designed research; AM, MD, HV, OA and YR performed the experiments and analyzed data; DE, OA, and YR wrote the paper. All authors read, provided feedback on, and approved the manuscript for publication. MD and HV equally contributed to the work.

## Funding

This work was supported by NIH grant R01 GM073857 (YR, OA and DE).

## Acknowledgments

The authors are grateful for the support of Dr. Sonia Sequeira for discussions, as well as members of the URI Institutional Development Award (IDeA) Network for Biomedical Research Excellence supported by NIH P20 GM103430, and Stryker Endoscopy (San Jose, CA) for providing their imaging system.

## Conflict of interest

DE, OA, and YR are founders of pHLIP, Inc., and they have shares in the company. pHLIP, Inc provided funding for synthesis and purification of modified STINGa and ICG-pHLIP, and the FACS analysis performed at the Charles River Discovery Labs.

The IP for pHLIP-STINGa was exclusively licensed to pHLIP, Inc.

The remaining authors declare that the research was conducted in the absence of any commercial or financial relationships that could be construed as a potential conflict of interest.

The handling editor PM declared a shared affiliation with the author DE at the time of review.

## Publisher’s note

All claims expressed in this article are solely those of the authors and do not necessarily represent those of their affiliated organizations, or those of the publisher, the editors and the reviewers. Any product that may be evaluated in this article, or claim that may be made by its manufacturer, is not guaranteed or endorsed by the publisher.
