# Supplementary figures and images for: Eradication of tumors and development of anti-cancer immunity using STINGa targeted by pHLIP
Source: Front Oncol. 2022 Oct 18;12:1023959. doi: 10.3389/fonc.2022.1023959 (PMC9622777; doi:10.3389/fonc.2022.1023959)

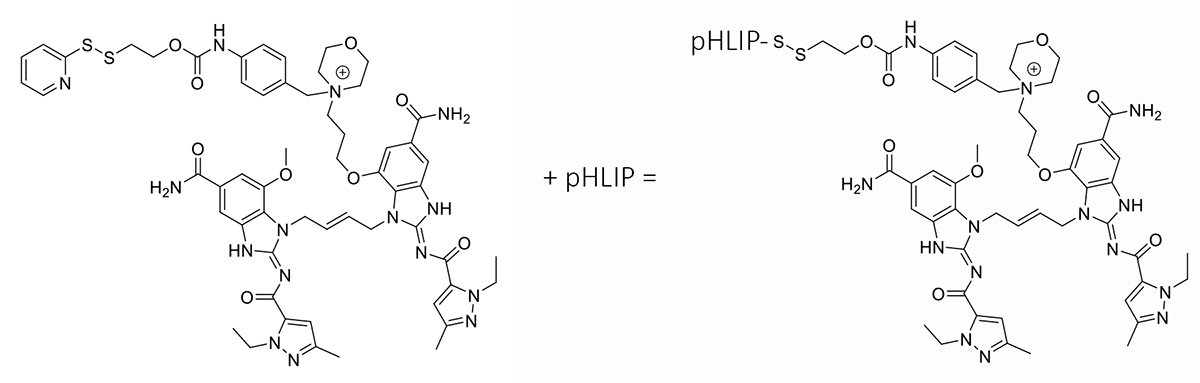

Supplement: Supplementary file 1 [file Image_1.jpg]

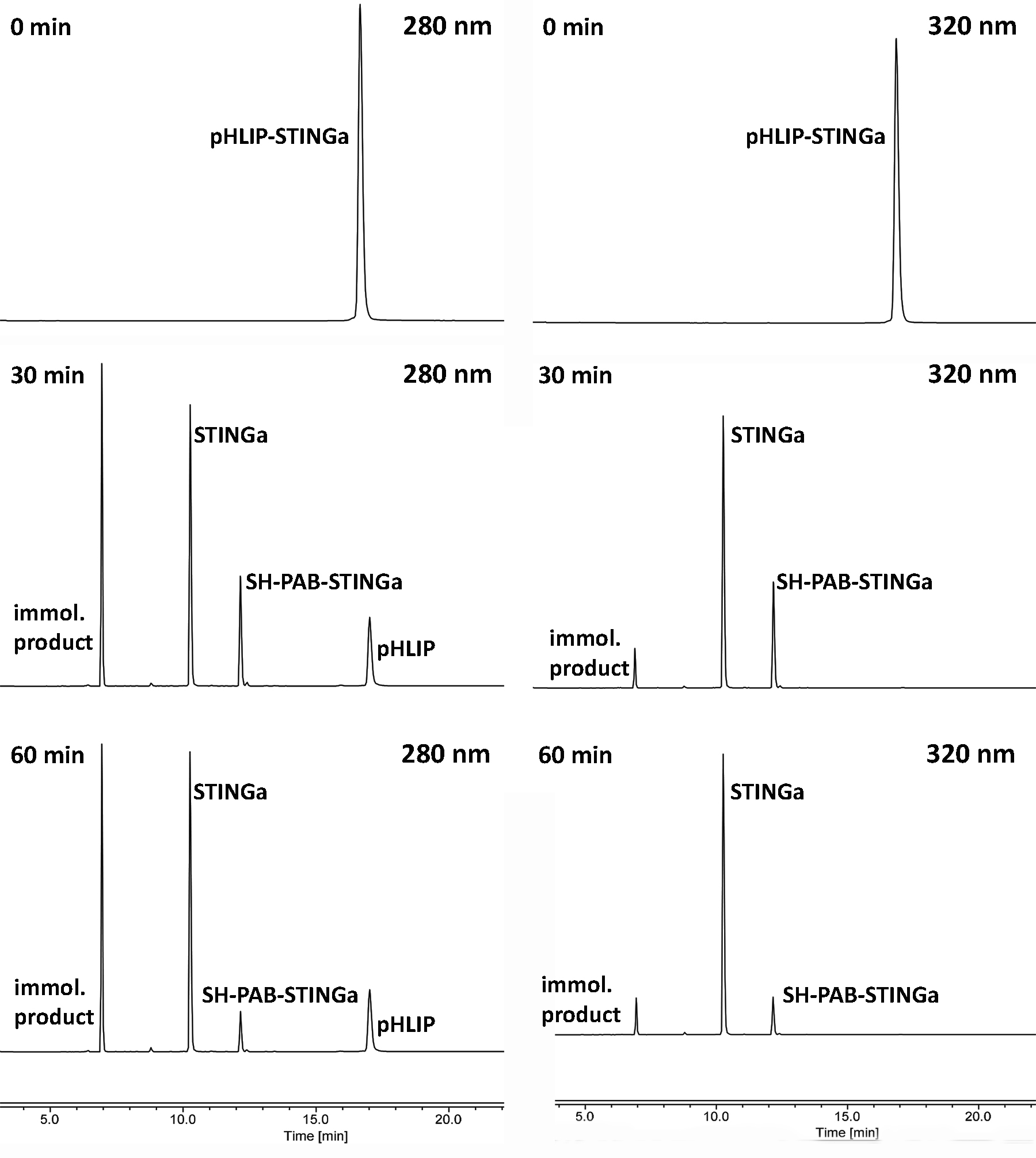

Supplement: Supplementary file 2 [file Image_2.jpg]

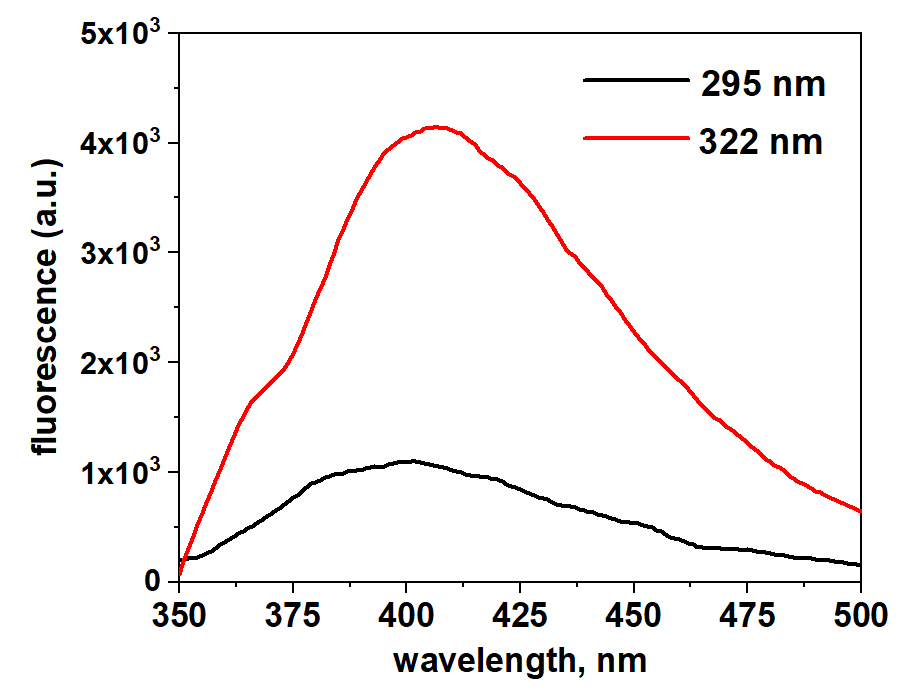

Supplement: Supplementary file 3 [file Image_3.jpg]

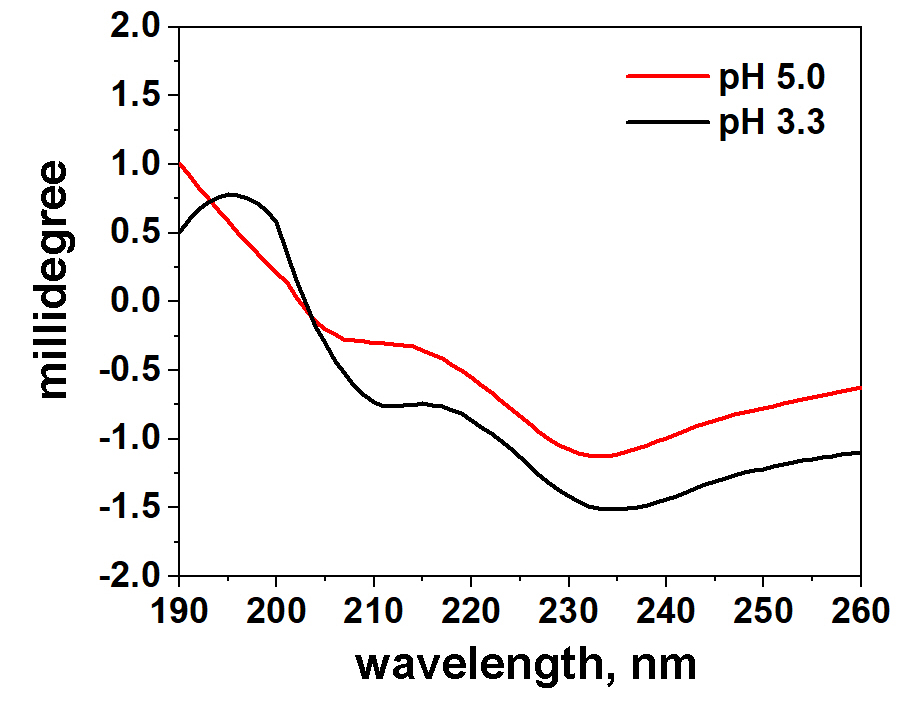

Supplement: Supplementary file 4 [file Image_4.jpg]

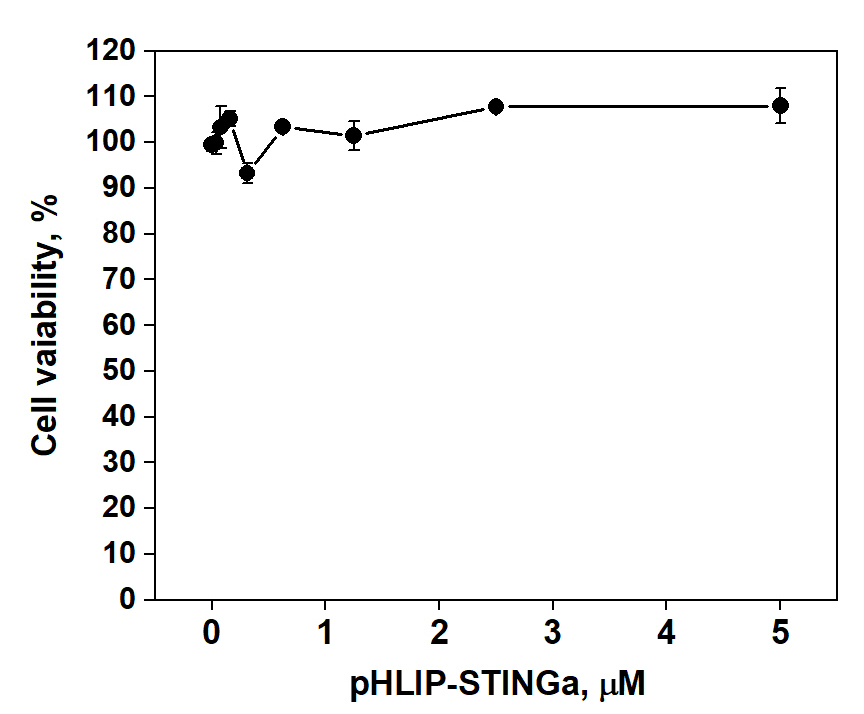

Supplement: Supplementary file 5 [file Image_5.jpg]

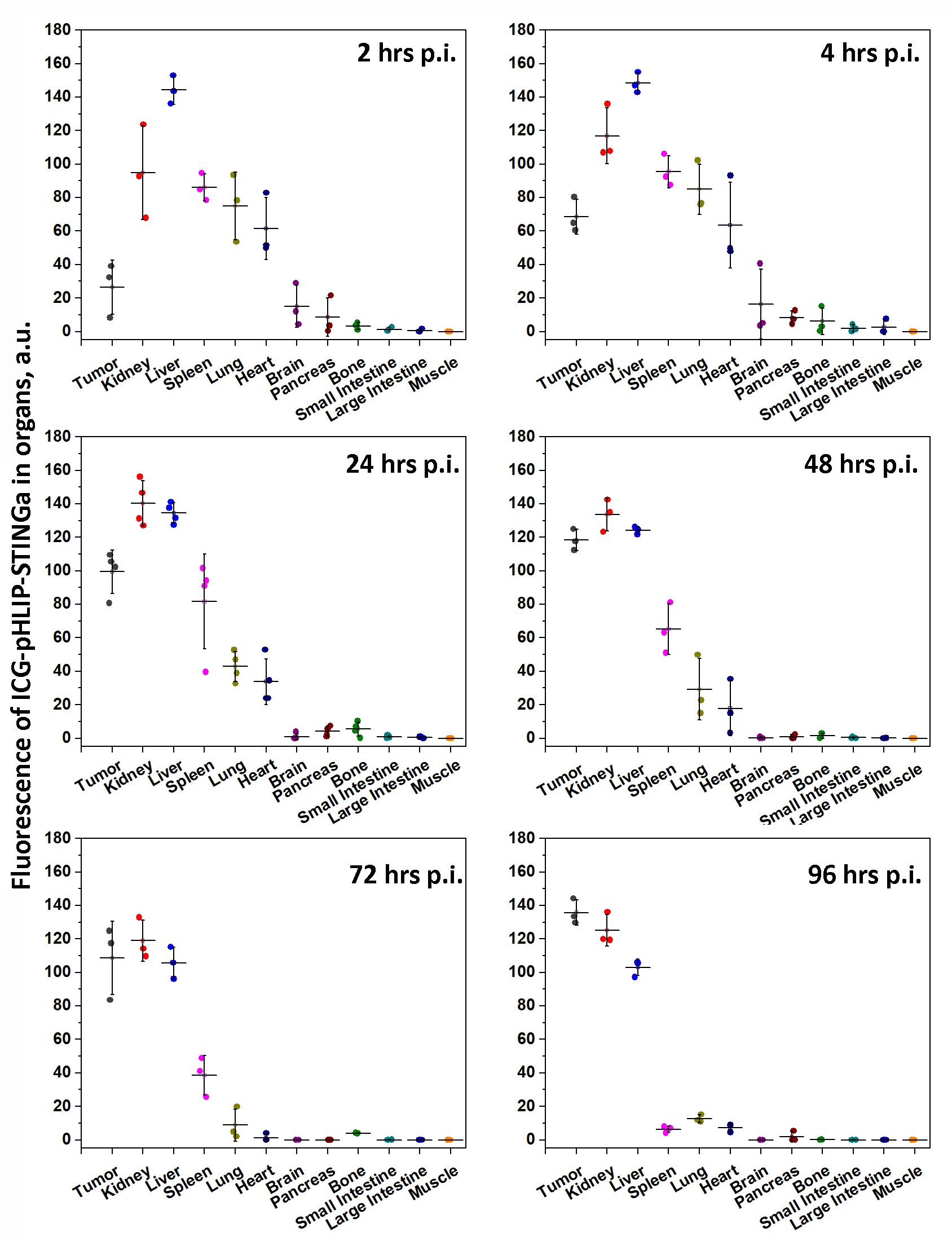

Supplement: Supplementary file 6 [file Image_6.jpg]

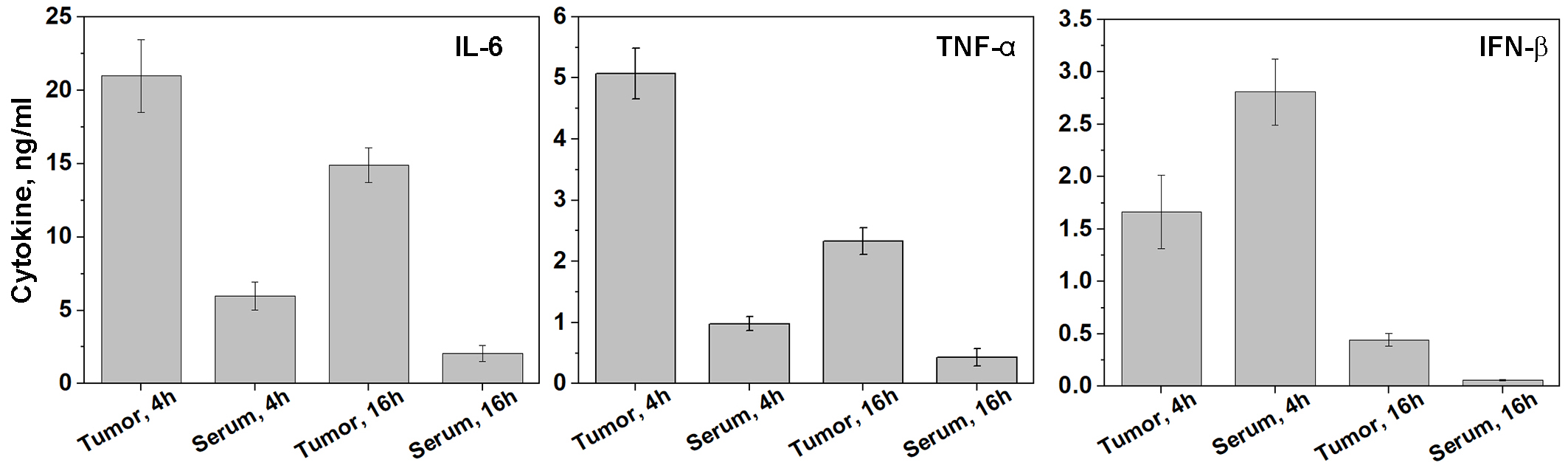

Supplement: Supplementary file 7 [file Image_7.jpg]

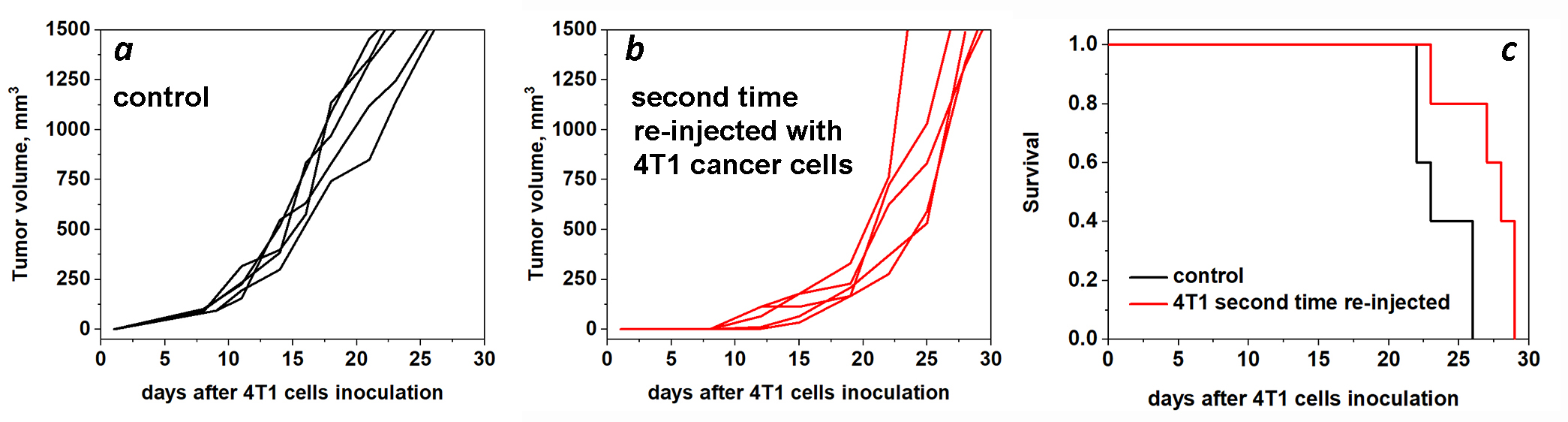

Supplement: Supplementary file 8 [file Image_8.jpg]

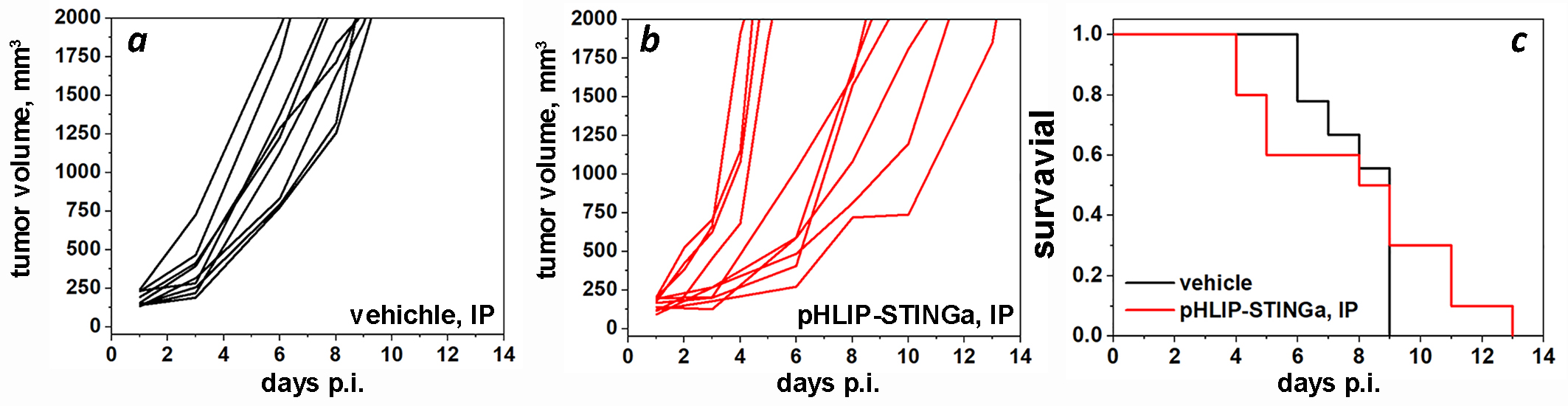

Supplement: Supplementary file 9 [file Image_9.jpg]

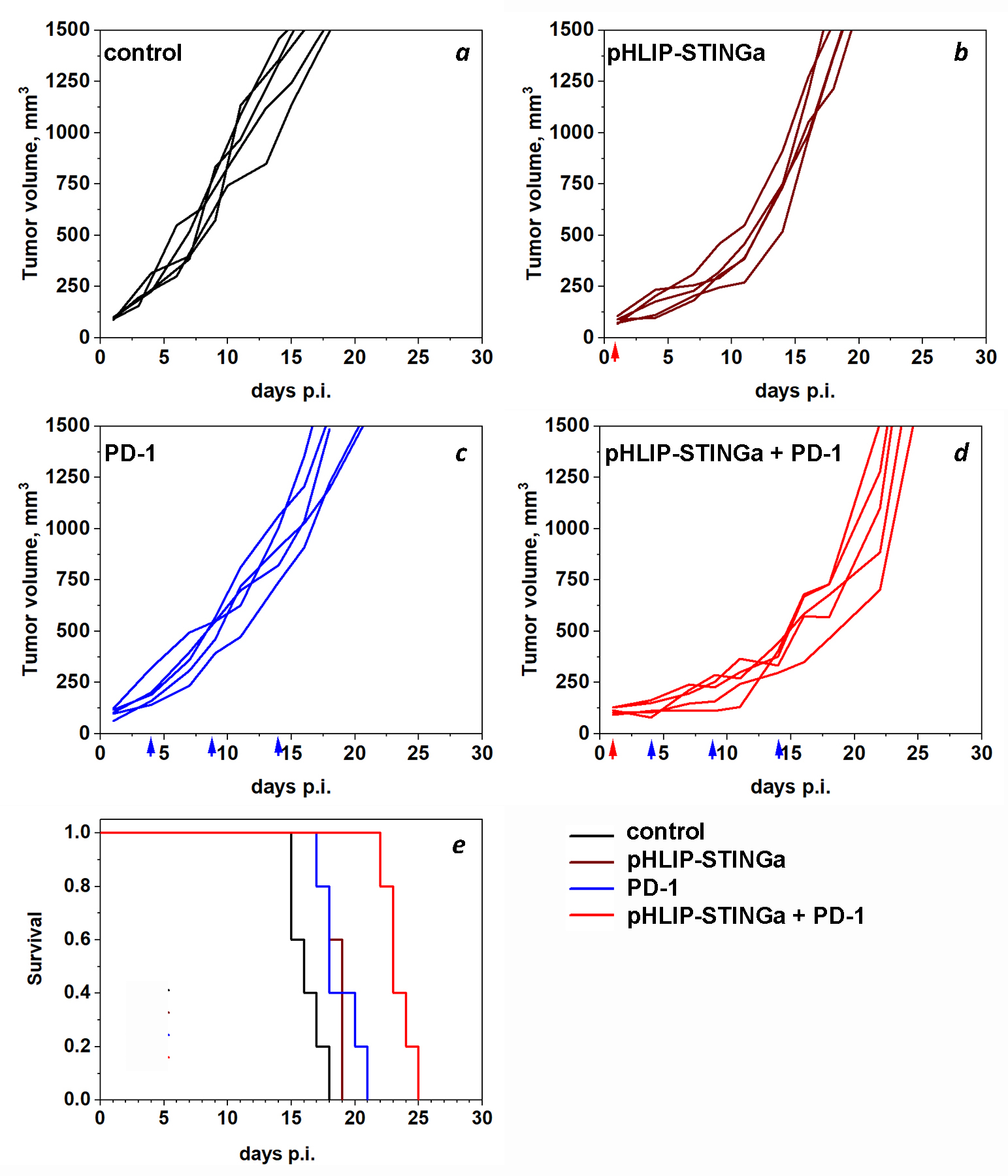

Supplement: Supplementary file 10 [file Image_10.jpg]

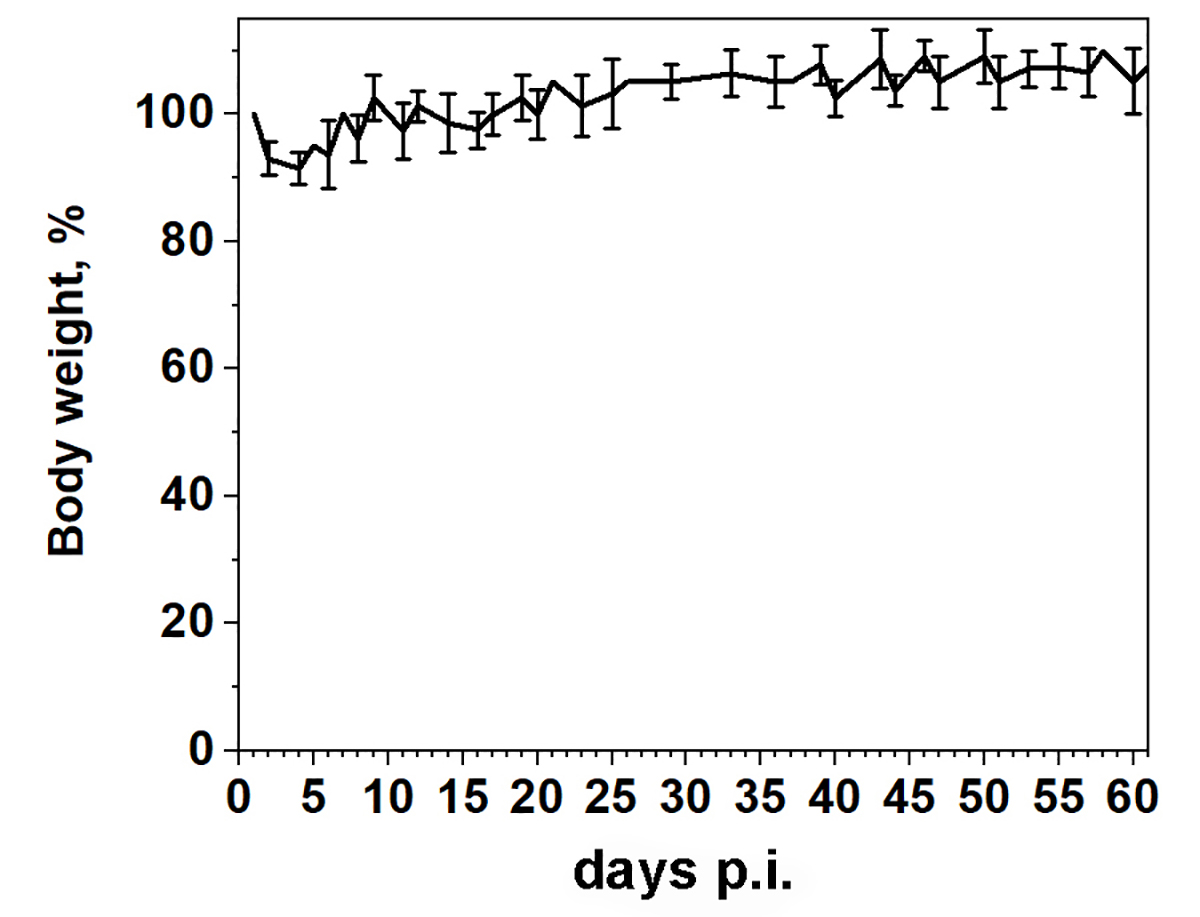

Supplement: Supplementary file 11 [file Image_11.jpg]

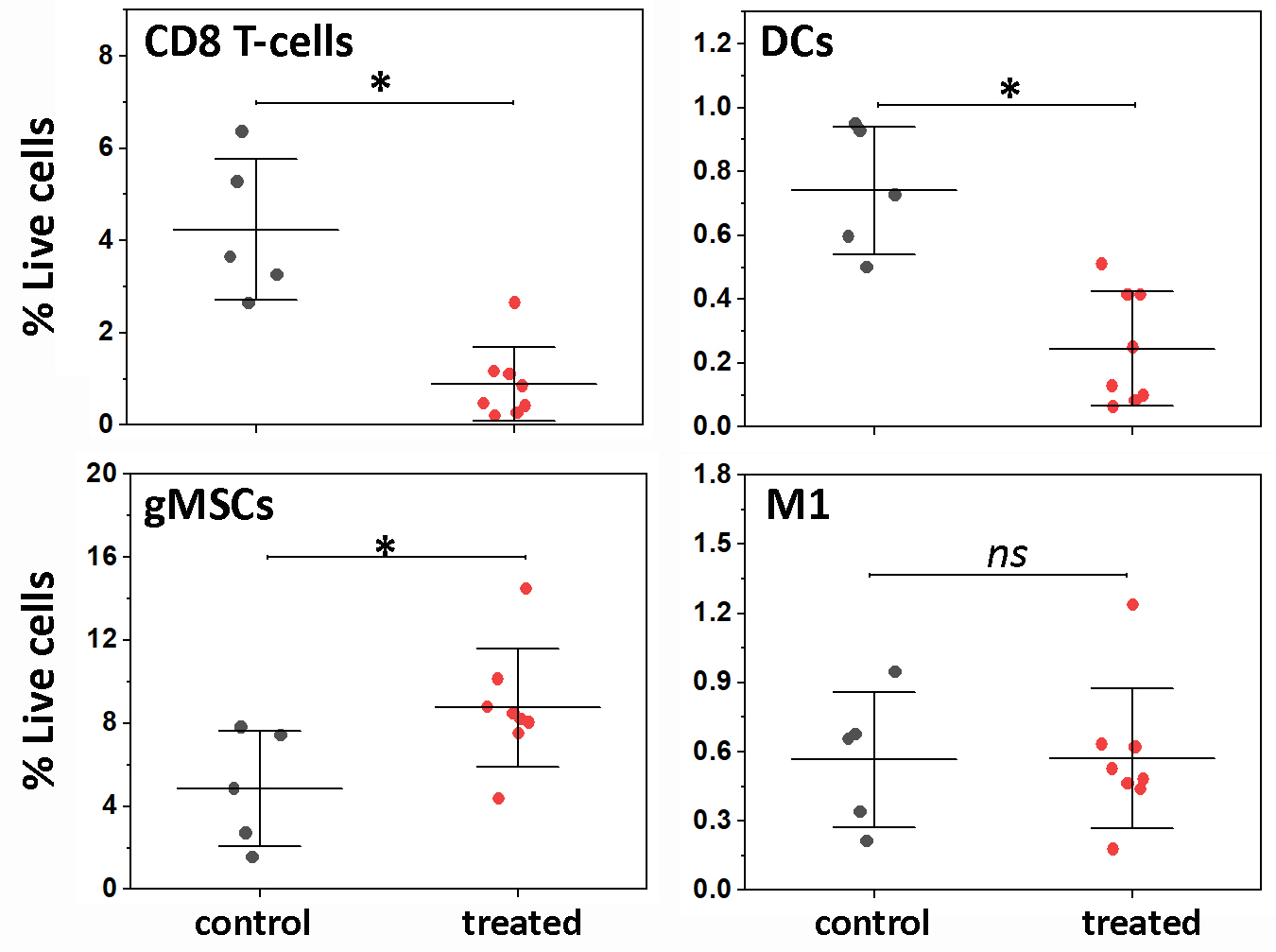

Supplement: Supplementary file 12 [file Image_12.jpg]

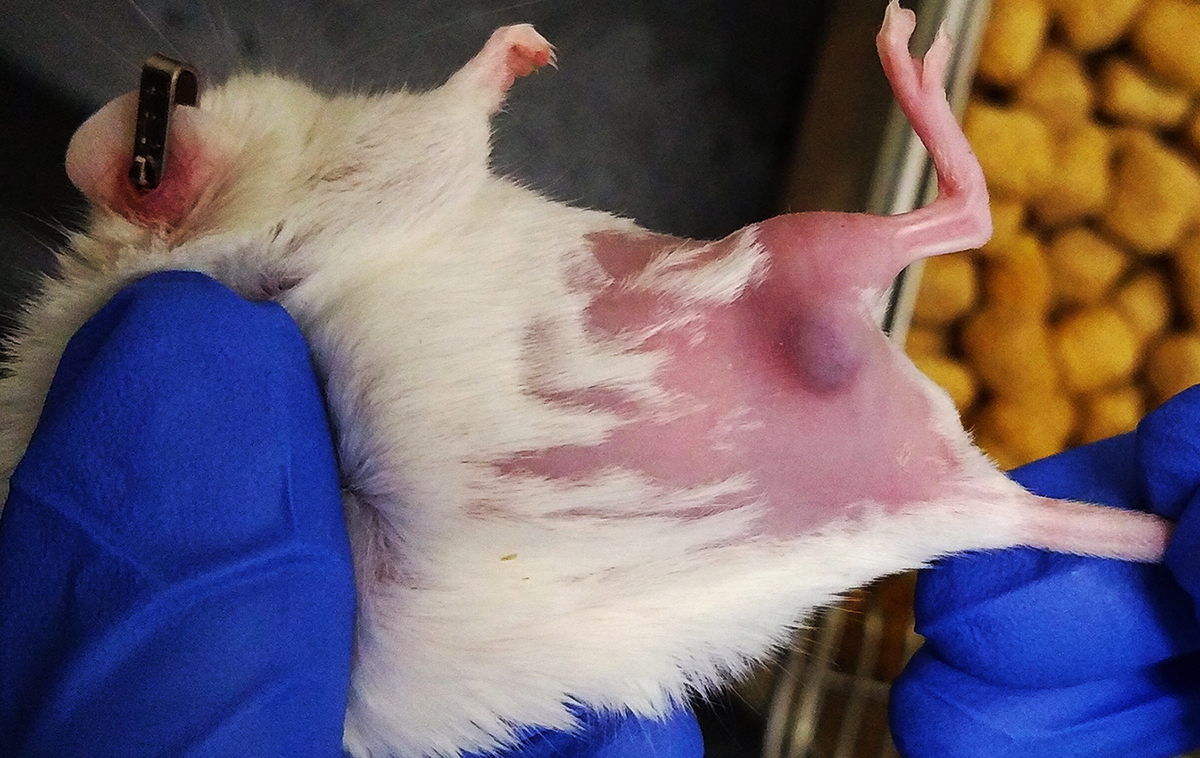

Supplement: Supplementary file 13 [file Image_13.jpg]
