# Supplementary material for: Eradication of tumors and development of anti-cancer immunity using STINGa targeted by pHLIP
Source: Front Oncol. 2022 Oct 18;12:1023959. doi: 10.3389/fonc.2022.1023959 (PMC9622777; doi:10.3389/fonc.2022.1023959)
Supplement: Supplementary file 14 [file Table_1.docx]

**Table S1. Stability in mouse and human plasma.** The amount (in %) of the undegraded pHLIP-STINGa products are presented as their free fractions (unbound to mouse or human plasma proteins) at different time points of incubation. The amounts (in %) of pHLIP-STINGa products bound to mouse or human plasma proteins are shown in brackets.

| **Time, h** | **pHLIP(Laa)-STINGa** | | **pHLIP(Daa)-STINGa** | |
| --- | --- | --- | --- | --- |
|  | **mouse** | **human** | **mouse** | **human** |
| 0 | 99.8 (0) | | 99.4 (0) | |
| 2 | 90.0 (5.5) | 95.1 (3.9) | 90.9 (51.7) | 99.2 (77.2) |
| 4 | 82.7 (16.3) | 91.4 (5.3) | 84.5 (59.6) | 97.6 (84.4) |
| 24 | 57.0 (33.1) | 64.5 (20.5) | 58.2 (75.9) | 89.6 (87.7) |
