# Supplementary material for: Eradication of tumors and development of anti-cancer immunity using STINGa targeted by pHLIP
Source: Front Oncol. 2022 Oct 18;12:1023959. doi: 10.3389/fonc.2022.1023959 (PMC9622777; doi:10.3389/fonc.2022.1023959)
Supplement: Supplementary file 15 [file DataSheet_1.docx]

**Supplementary Figure Legends**

**Figure S1. Chemical structure of pHLIP-STINGa.** STINGa (diABZI) was modified for conjugation with a single Cys residue at the C-terminal ends of pHLIP peptides to obtain S-S cleavable, self-immolating pHLIP-STINGa.

**Figure S2. Kinetics of self-immolation**. HPLC chromatograms recorded at 280 nm and 320 nm for pHLIP-STINGa treated with DTT dissolved in 50 mM phosphate buffer containing 150 mM NaCl pH 7.4 at different time points (0, 30, 60 min) after addition of DTT are shown. Retention times are the following pHLIP-STINGa: 16.78 min, pHLIP: 17.02 min (has no signal at 320 nm), SH-PAB-STINGa (modified diABZI cleaved from pHLIP): 12.16 min, STINGa (original diABZI): 10.27 min, product of immolation: 6.95 min.

**Figure S3. DiABZI fluorescence.** Fluorescence spectra of STINGa (diABZI) in phosphate buffer excited at 295 nm and 322 nm are shown.

**Figure S4. OCD spectra.** OCD spectra were recorded 12 hours after deposition of solution of pHLIP-STINGa and POPC in supported layers at pH 5 and pH 3.3

**Figure S5. Cell viability**. THP1 cells were activated and polarized into M2 macrophages by treatment with PMA for 6 hours followed by treatment with IL-4/IL-13 for another 18 hours. After activation and polarization all factors were removed and cells were treated with pHLIP-STINGa at pH 6.4 in DMEM without FBS for 3 hours. Then, FBS was added up to 10% and pH increased to pH 7.4 and cells were incubated for 48 hours after which MTS calorimetric assay was performed.

**Figure S6. Biodistribution of ICG-pHLIP-STINGa in organs and tissues.** Mean fluorescence per area was calculated for each tissue and organ at 2, 4, 24, 48, 72 and 96 hours post-injection after a single IV injection of ICG-pHLIP-STINGa (200 μM 100 μl). All points, means and St.d. are shown.

**Figure S7.** **Average level of cytokines** (IL-6, TNF-α, IFN-β) in tumor supernatants and serum measured by ELISA at 4 and 16 hrs after a single IV (200 μM 150 μl) or IP (100 μM 300 μl) injections of pHLIP(Laa)-STINGa or pHLIP(Daa)-STINGa.

**Figure S8. Immune memory was not developed for 4T1 cancer cells.** 4T1 tumors growth curves in “control” group (***a***) and “second time re-injected with 4T1 cancer cells” group (***b***) are shown. Mice in control group received 10^5^ 4T1 cancer cells in right flank. Mice in the second time re-injected group were first inoculated with 5x10^4^ CT26 cancer cells. When tumors reached about 100 mm^3^ in volume mice received a single IP injection of pHLIP-STINGa (100 μM 300 µl) on day 1, tumors were eradicated, then on day 61, 5x10^4^ CT26 cancer cells were inoculated in left flank, tumors did not develop, and, finally, on day 101 10^5^ 4T1 cancer cells were inoculated in right flank and 4T1 tumors were developed. ***c***) Kaplan-Meier survival plots obtained for data shown on panels ***a*** and ***b***.

**Figure S9. Treatment of CT26 tumors in athymic nude mice lacking T-cells.** ***a-b***) CT26 tumors growth curves in nude athymic mice are shown after a single IP injection of pHLIP-STINGa (100 μM 300 μl) or vehicle on day 1, when tumors reached about 100-200 mm^3^ in volume. ***c***) Kaplan-Meier survival plots obtained for data shown on panels ***a-b***.

**Figure S10. Treatment of 4T1 tumors.** 10^5^ of 4T1 breast cancer cells were inoculated in right flank of Balb/C mice. When tumor reached about 100 mm^3^ in volume (day 1) a single IP injection of pHLIP-STINGa (100 μM 300 µl) was given to groups pHLIP-STINGa and pHLIP-STINGa + PD-1 (shown by red arrow), and 3 IP injections on days 4, 9 and 14 of PD-1antibody (250 μg per injection) were given to groups PD-1 and pHLIP-STINGa + PD-1 (shown by blue arrow). Control group did not receive treatment. Tumors growth of control (***a***), pHLIP-STINGa (***b***), PD-1 (***c***) and pHLIP-STINGa + PD-1 (***d***) are shown. ***e***) Kaplan-Meier survival plots based on the data shown on panels ***a-d*** are presented. The *p*-levels for statistical significance of improved survival of pHLIP-STINGa vs control and PD-1 vs control was established to be *p-*level < 0.02 calculated using Log rank (by weighting all time points the same), Breslow method (by weighting all time points by the number of cases at risk at each time point) and Tarone-Ware method (by weighting all time points by the square root of the number of cases at risk at each time point), and *p*< 0.004 for pHLIP-STINGa + PD-1 vs control.

**Figure S11. Body weight**. The change of body weight after a single IP injection of pHLIP-STINGa (100 µM 300 µl) received on day 1 is shown. Similar body weight curves were obtained for other routes of administrations in Balb/c and athymic nude mice.

**Figure S12. Population of immune cells in treated and control (non-treated) mice**. The numbers of cells within the TME in control, *n=5,* (mice receiving vehicle as a single IP injection) and treated, *n=8,* (mice receiving a single IP injection of Al647-pHLIP-STINGa (100 μM 300 μl)) were established by FACS analysis at 24 hours p.i.. The percentages of CD8 T-cells, (CD45^+^, CD3^+^, CD11b^-^, CD4^-^, CD8^+^), DCs (CD45^+^, CD11b^+^, CD11c^+^, MHCII^+^, F4/80^-^), gMDSCs (CD45^+^, CD3^-^, CD11b^+^, F4/80^-^, Ly6C^low^, Ly6G^+^) and M1-type macrophages (CD45^+^, CD11b^+^, F4/80^+^, CD206^-^) quantified as % of all live cells in control and treated groups are shown. The number of CD4 T-cells and Treg cells was less than 0.1% of all cells in both control and treated groups. All points, mean and St.D. are shown, and *p*-*level* was calculated using the Kolmogorov-Smirnov two-tailed nonparametric test ( means *p-level* < 0.05).

**Figure S13. Intra-tumoral hemorrhage** was observed within 2-4 days after pHLIP-STINGa injection. Image of Balb/c mice with CT26 tumor in right flank next day after a single IP injection of pHLIP-STINGa (300 µM 100 μl) is shown.
